# Impact of Negative Emotion on the Neural Correlates of Long-Term Recognition in Younger and Older Adults

**DOI:** 10.3389/fnint.2012.00074

**Published:** 2012-09-19

**Authors:** Grégoria Kalpouzos, Håkan Fischer, Anna Rieckmann, Stuart W. S. MacDonald, Lars Bäckman

**Affiliations:** ^1^Aging Research Center, Karolinska Institute and Stockholm UniversityStockholm, Sweden; ^2^Department of Psychology, Stockholm UniversityStockholm, Sweden; ^3^Department of Psychology, University of VictoriaVictoria, BC, Canada

**Keywords:** aging, amygdala, emotion, episodic memory, hippocampus, long-term memory, prefrontal cortex, recognition

## Abstract

Some studies have suggested that the memory advantage for negative emotional information over neutral information (“negativity effect”) is reduced in aging. Besides the fact that most findings are based on immediate retrieval, the neural underpinnings of long-term emotional memory in aging have so far not been investigated. To address these issues, we assessed recognition of neutral and negative scenes after 1- and 3-week retention intervals in younger and older adults using functional magnetic resonance imaging. We further used an event-related design in order to disentangle successful, false, and true recognition. This study revealed four key findings: (1) increased retention interval induced an increased rate of false recognitions for negative scenes, canceling out the negativity effect (present for hit rates only) on discrimination in both younger and older adults; (2) in younger, but not older, adults, reduced activity of the medial temporal lobe was observed over time for neutral scenes, but not for negative scenes, where stable or increased activity was seen; (3) engagement of amygdala (AMG) was observed in older adults after a 3-week delay during successful recognition of negative scenes (hits vs. misses) in comparison with neutral scenes, which may indicate engagement of automatic processes, but engagement of ventrolateral prefrontal cortex was unrelated to AMG activity and performance; and (4) after 3 weeks, but not after 1 week, true recognition of negative scenes was characterized by more activity in left hippocampus and lateral occipito-temporal regions (hits vs. false alarms). As these regions are known to be related to consolidation mechanisms, the observed pattern may indicate the presence of delayed consolidation of true memories. Nonetheless, older adults’ low performance in discrimination of negative scenes could reflect the fact that overall, after long delays of retention, they rely more on general information rather than on perceptual detail in making recognition judgments.

## Introduction

Emotion is closely linked to memory because of its importance for survival (e.g., remembering that a previously experienced situation was life-threatening). Hence, negative emotions may enhance memory retrieval and be resistant to forgetting (Weymar et al., 2011). Numerous studies have demonstrated better recognition of negative compared to neutral information in young adults (Kensinger, 2007). This effect may partly reflect reduced forgetting via enhanced consolidation mechanisms (LaBar and Phelps, 1998; Sharot and Phelps, 2004; Sharot and Yonelinas, 2008; Pierce and Kensinger, 2011). This “negativity effect” has been linked to engagement of the amygdala (AMG) in association with the hippocampus (HC) during processing of emotional information, notably at encoding (see Murty et al., 2010; Sabatinelli et al., 2011, for meta-analyses, and Dolcos et al., 2012 for a recent review). Fewer studies have investigated the neural underpinnings of emotional retrieval, especially after long retention intervals. Yet, these studies showed specific or more AMG activity during retrieval of emotional compared to neutral information, for retention intervals between 5 min and 1 year (Dolan et al., 2000; Dolcos et al., 2005; Kensinger and Schacter, 2005, 2007; Keightley et al., 2011).

Socioemotional selectivity theory postulates increased emotion regulation with advancing adult age. This assertion is based on the perception of remaining lifetime driving motivations and goals (e.g., Carstensen et al., 1999). Some research reveals a “positivity effect,” where positive events are enhanced and therefore better remembered, along with a reduced “negativity effect,” where negative events are more likely to be forgotten in older adults (Charles et al., 2003; Mather and Carstensen, 2005; Mather, 2008, 2012; Spaniol et al., 2008; Reed and Carstensen, 2012). However, while findings regarding the positivity effect converge toward a preservation of this effect in aging, results concerning the negativity effect are mixed, as several studies show a memory advantage of negative over neutral information also in older adults (Kensinger et al., 2002; Denburg et al., 2003; Otani et al., 2007; Murty et al., 2009; Gavazzeni et al., 2012). To our knowledge, only Waring and Kensinger (2009) have tested the effect of retention interval on emotional memory in aging. Despite a general age-related decrease in recognition accuracy, both younger and older adults demonstrated similar memory enhancement for positive and negative scenes. Moreover, this effect was most pronounced for the longest retention interval (1 day vs. a few minutes), suggesting preserved consolidation mechanisms for emotional informating in aging, at least over 1 day of retention.

Relatively little is known about how the brain processes emotional information in old age (see also Ebner et al., 2012; Pollock et al., 2012). In the following, we review findings from four studies. In all of them, older participants showed a negativity effect, inconsistent with the Socioemotional selectivity theory. Both St Jacques et al. (2009b) and Kensinger and Schacter (2008) showed that AMG was engaged in both younger and older adults during successful encoding of negative pictures. In St Jacques et al., a hemispheric age-related difference was observed: there was greater activity in left AMG in the younger group, and greater activity in right AMG in the older group. By contrast, Fischer et al. (2010) reported age-related differences in brain activity during successful encoding of fearful faces: more AMG and HC activity was found in the younger group, and more activity in the right prefrontal cortex (PFC) was observed in the older group. Murty et al. (2009) scanned younger and older adults during emotional retrieval. Similar to Fischer et al. (2010), the young exhibited more AMG activity, whereas the old showed more PFC activity. Overall, the emerging pattern from these studies is an age-related reduction of AMG activity coupled with increased PFC activity during encoding and retrieval of emotional information. Some authors have interpreted this shift in patterns of activity as compensatory (Murty et al., 2009), and others have argued that it reflects increased regulation of emotional processes with age-related differences in AMG-PFC functional connectivity (for review, see St Jacques et al., 2009a). However, these interpretations are based on encoding data mostly, while Murty et al.’s encoding-retrieval study was a blocked design, showing therefore sustained rather than transient brain activity, the latter allowing to investigate *successful* retrieval. Moreover, in the aforementioned studies, the retrieval session took place from 2 to 45 min after the encoding session, precluding conclusions about neural substrates of long-term emotional memory in aging. Hence, in the present study we sought to uncover transient neural activity during emotional long-term retrieval in aging.

Fischer et al. (2010) noted an important feature of aging effects on recognition memory, namely the tendency to produce more false recognitions of negative emotional items among older adults. Emotionality may result in more false alarms (FA), because of greater semantic cohesiveness of emotional items (e.g., emotional items tend to belong to semantic categories in which instances share more features than for neutral items; Maratos et al., 2000; Marchewka et al., 2008). In this case, individuals may base their recognition decision on general characteristics, or in other words on familiarity processes. Conversely, others have argued that emotionality may reduce false memories, because emotion increases item distinctiveness (Kensinger and Corkin, 2004; for review, see Kensinger, 2012). In this case, recognition would be based on recollection mechanisms (e.g., recognition of specific features of the items). Thus, findings are mixed and the studies in question used short retention intervals. In a study of younger adults, Howe et al. (2010) found a significant increase of false recognitions for negative, but not for neutral, items from immediate to 1-week retrieval. Older adults tend to rely more on familiarity than on recollection in making recognition judgments (Bastin and van der Linden, 2003; Howard et al., 2006; Prull et al., 2006), which is known to increase false recognitions. Thus, the increase of false recognitions of negative items over time observed by Howe et al. in young adults may be exacerbated in older age, which may contribute to a reduced negativity effect in true recognition (discrimination) after long retention intervals.

In this study, younger and older adults underwent functional Magnetic Resonance Imaging (fMRI) during recognition of negative and neutral scenes at three occasions over 3 weeks. Here, we present behavioral and neural findings associated with recognition of negative and neutral scenes after 1 and 3 weeks of retention in relation to age. We were particularly interested in uncovering the effect of retention interval on the effect of negative emotion on memory in the presence of lures. The elaboration of an event-related fMRI design allowed us to study brain activity associated with successful, false, and true recognition.

## Materials and Methods

### Participants

Twenty younger and 20 older adults, all right-handed, were recruited. No subject reported any previous or current psychiatric, neurological or medical disease, and none was taking psychoactive medication or abused any substances. They were paid 1,500 SEK for their participation. The study was approved and conducted in accordance with guidelines established by the regional ethics committee, and written consent was obtained from all participants prior to the start of the study. All subjects initially underwent a cognitive battery. Their performance indicated that they were representative of their age cohorts, with typical negative age differences in fluid cognitive tasks along with no age differences in crystallized cognitive tasks. Because of misunderstanding of the MRI task (*n* = 2), technical issues (*n* = 1), MR findings (*n* = 1), or too few events to be analyzed in the fMRI data (*n* = 2), 19 young and 15 old subjects were retained for analyses. Their characteristics and cognitive scores are shown in Table 1.

**Table 1:** **Participant characteristics**.

	Young	Old	*t*-Tests
Number of participants (women)	19 (9)	15 (7)	
Age	25 ± 3.0	68.3 ± 2.6	
Age range	20–30	65–71	
Years of education	15.8 ± 1.8	14.2 ± 3.6*	*p* = 0.11
Vocabulary (synonym task, max = 30)	24.7 ± 2.9	26.4 ± 2.6	*p* = 0.09
Associative memory (verbal paired associates)			
Strong associates (max = 18)	16 ± 2.1	15.3 ± 1.9	*p* = 0.35
Weak associates (max = 18)	13.1 ± 3.8	7.7 ± 3.4	*p* < 0.001
Free recall (max = 16)	10.6 ± 2.7	6.6 ± 2.3	*p* < 0.001
Verbal fluency			
Category (fruits + clothes)	34.8 ± 6.7	34.5 ± 7.2	*p* = 0.89
Letter (A + F)	31.5 ± 10.9	30.1 ± 5.0	*p* = 0.63
Perceptual speed			
Figure comparison (max = 30)	21.7 ± 2.4	15.1 ± 2.9	*p* < 0.001
Letter comparison (max = 20)	9.9 ± 2.9	7.2 ± 3.3	*p* = 0.02
Working memory *n*-back			
2-Back (max = 10)	9 ± 0.8	6.6 ± 2.1	*p* < 0.001
3-Back (max = 9)	6.8 ± 1.1	4.4 ± 2.5	*p* < 0.001

Mean ± SD; *one missing value.

### General procedure

**Figure 1:**
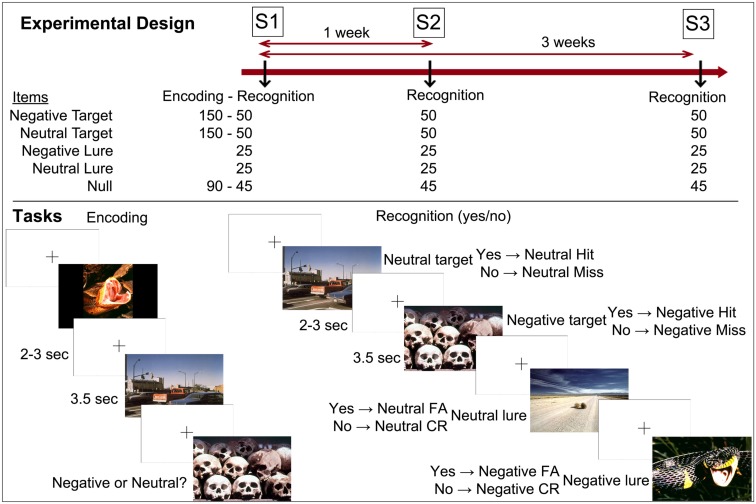
**Experimental design and tasks**. Notes: S1 (Session 1) = immediate recognition; S2 (Session 2) = 1-week delay recognition; S3 (Session 3) = 3-week delay recognition. CR, correct rejection; FA, false alarm.

The emotional memory task took place at three time points. At Session 1 (S1), subjects underwent intentional encoding of the material and immediate recognition. One week later, they came back and underwent the second recognition session (S2). Three weeks after S1 they undertook the third recognition session (S3). Subjects were scanned at all retrieval sessions (Figure 1).

### Materials and tasks

The task was displayed by means of E-prime (Psychology Software Tools Inc., Pittsburgh, PA, USA) using an IBM ThinkPad 390 computer (IBM, San Jose, CA, USA). The pictures were projected onto a screen in the scanner room using a Philips Hoper HG 20 Impact LCD projector (Philips Corp., Eindhoven, The Netherlands), positioned approximately 3 m in front of the scanner. Stimuli were presented via a mirror system, which was placed on top of the head coil 2–5 cm from the subjects’ eyes.

The material consisted of 450 pictures selected from the International Affective Picture System (IAPS, Lang et al., 2008). At encoding, 150 neutral and 150 negative pictures were presented, together with 90 null events (three black crosses on a white background with button press). Subjects were told to memorize the scenes for later retrieval, and to rate the valence of the pictures as neutral or negative. Fifty pictures of each type (neutral, negative) were shown as targets at each recognition session together with 25 lures of each type and 45 null events. The three types of events (targets, lures, null events) were inter-mixed. The order of events was identical for all subjects in order to control for between-person order effects. The pictures were presented during 3.5 s each, and were separated by a cross (on a white background) that lasted for 2–3 s. Participants responded by button presses to indicate whether or not pictures were shown during encoding (yes/no recognition). Overall, negative scenes were more arousing than neutral scenes, and these two categories (negative, neutral) also differed according to the living/non-living dimension. Importantly, within each emotional category of items, the sets of scenes did not differ on valence, arousal, and living/non-living dimensions across sessions of retrieval (no main effect of session and no interaction between emotion and session on valence, arousal, and living/non-living dimensions). Although lures were more arousing than targets, this should not have had any effect on findings as this factor did not interact with emotion or session of retrieval (Table A1 in Appendix). The material was distributed and balanced across the retrieval sessions to reach the aforementioned criteria according to normative data and results from a pilot study.

### Neuroimaging

The study was carried out on a GE 1.5 T MRI scanner using an eight-channel head coil. For the event-related functional scanning, an EPI (echo planar imaging) sequence was used with the following parameters: Repetition time (TR) = 2,500 ms (32 axial slices acquired in an interleaved order), echo time (TE) = 40 ms, flip angle = 90°, field of view (FOV) = 22 cm, slice thickness = 4.5 mm, in-plane resolution = 3.4 mm × 3.4 mm, interslice spacing = 0.5 mm. To avoid signals arising from progressive saturation, four dummy scans were performed prior to image acquisition. Each recognition task (S1, S2, and S3) was run within one session of 19.5 min, resulting in 482 EPI volumes per session. Structural T1-weighted images were also collected with the following parameters: TR = 24 ms (124 coronal slices acquired), TE = 6 ms, flip angle = 35°, FOV = 22 cm, slice thickness = 1.5 mm, in-plane resolution = 0.86 mm × 0.86 mm, no gap.

### Analyses

#### Behavioral data

Hits (H) and FA were calculated following the procedure described by Snodgrass and Corwin (1988), where a correction was applied by adding +0.5 to H (and FA) and +1 to the number of targets (and lures), e.g., H = (*N* hits + 0.5)/(*N* targets + 1). Discrimination was calculated by subtracting FA from H. Analyses of variance were conducted on Hits, FA, and Discrimination with the factors Age (Younger, Older), Session (S1, S2, S3), and Emotion (Neutral, Negative).

#### Neuroimaging data

SPM8 (Statistical Parametric Mapping, Wellcome Trust Centre for Neuroimaging, London)^1^ implemented in Matlab 7.13 (Mathworks, Inc., MA, USA) was used to analyze the imaging data.

##### Preprocessing

The structural T1 images were aligned to the standard MNI template (Montreal Neurological Institute) and voxel-based morphometry was run using the VBM8 toolbox of SPM8 (with DARTEL spatial normalization, Ashburner, 2007), using the default parameters. The normalized gray matter (GM) images were used for volumetric assessment (see Results and HC and AMG Age-Related Atrophy). Furthermore, a binary GM mask was built for use as an explicit mask in the fMRI analyses: using the ImCalc function of SPM, an average GM image was created from the individual normalized GM images of all participants, and this image was transformed into a binary mask. We also built two group-specific GM masks for within-group analyses (younger, older).

The fMRI dataset was preprocessed using the following steps: first correction for differences in slice timing was applied within each EPI volume (the middle slice was the reference slice), then the images were spatially realigned (the mean EPI image created by SPM was co-registered to the corresponding T1 image, and all volumes were realigned to the mean EPI image) and unwarped. The images were then spatially normalized to the MNI template, resampled to a voxel size of 2 mm^3^, and smoothed with an 8-mm full-width at half-maximum Gaussian filter kernel.

##### Statistics

For the fMRI data, single-subject contrasts were set up using the general linear model and group data were analyzed with a random-effects model. All models were convolved with a canonical hemodynamic response function as implemented in SPM. Ten regressors were constructed in order to capture the variance of all events (see bottom panel of Figure 1, showing the eight regressors of interest, to which two other regressors were added: baseline and no responses). All events were modeled as delta functions. Covariates of no interest included the six realignment parameters to further account for signal-changes related to inadvertent head motion. Subject analyses were run separately for each retrieval session.

At the group level, statistical parametric maps were generated voxel by voxel using factorial designs. The contrast of interest used to assess brain areas whose activity was related to successful recognition was “H − Misses (M).” The data of Session 1 were not analyzed because of too few M; therefore only the data of S2 and S3 were used in this study. Although the design of the study contains three factors, we conducted separate factorial designs with two factors each as follows: (1) two within-age-group ANOVAs were conducted with the factors Session (S2, S3) and Emotion (Neutral, Negative); (2) two within-session ANOVAs were conducted with the factors Age (Younger, Older) and Emotion (Neutral, Negative); and (3) two within-emotional-valence ANOVAs were conducted with the factors Session (S2, S3) and Age (Younger, Older). This decision was based on the fact that contrasts that do not span all conditions (or cells) at the second-level analyses are not recommended in multifactorial designs. For instance, if the three-way ANOVA was carried out and the interaction of interest (e.g., Emotion × Session) lied within the younger but not the older group, the sphericity assumption (i.e., pooled variance across all factors) may not be fulfilled. Hence, conducting two-way ANOVAs allowed testing all potential interactions without ruling out the sphericity assumption.

Brain areas related to false recognitions were assessed in the older group and only for negative items at S2 and S3. This restriction to the older group and to negative items was due to the number of events: Too few events of this type occurred in the younger group and overall in the neutral condition, precluding meaningful analyses. One older adult produced only four FA at S2 but we decided to keep this subject in the analyses (his presence did not alter the pattern of findings and he was not an outlier at the neuroimaging level). To fathom the neural basis of false recognition of negative scenes, we computed *t*-tests using the contrasts FA vs. correct rejections (CR) and H vs. FA separately for S2 and S3. Therefore, we distinguished the terms “successful recognition” and “true recognition.” Successful recognition was assessed with the contrast “H vs. M,” regardless of the cognitive processes or confidence with which the subjects made their recognition decision. At the cognitive level, this contrast corresponds to Hits only. True recognition was assessed with the contrast “H vs. FA”; given the proportion of FA especially in the older adults for the negative items (see Results), it is likely that many negative Hit responses were made based on the same processes or confidence as for negative FA – thus, removing FA-related brain activity from Hit-related activity may show brain areas whose activity was related to true recognition. At the cognitive level, this contrast corresponds to discrimination.

At the voxel level, we used an uncorrected threshold of *p* < 0.001 (cluster threshold *k* > 20 contiguous voxels). For the *a priori* HC (left, right) and AMG (left, right) regions of interest (ROIs) we used a threshold of *p* < 0.0125 (i.e., *p* < 0.05 corrected for four ROIs, and *k* > 20). ROIs were taken from the WFU Pickatlas (Maldjian et al., 2003) and the AAL atlas (Tzourio-Mazoyer et al., 2002). Contrast values as displayed on the graphs were extracted from the clusters of interest (over all voxels of the given clusters) using the eigenvariate tool in SPM.

## Results

### Behavioral data

#### Hits

**Figure 2:**
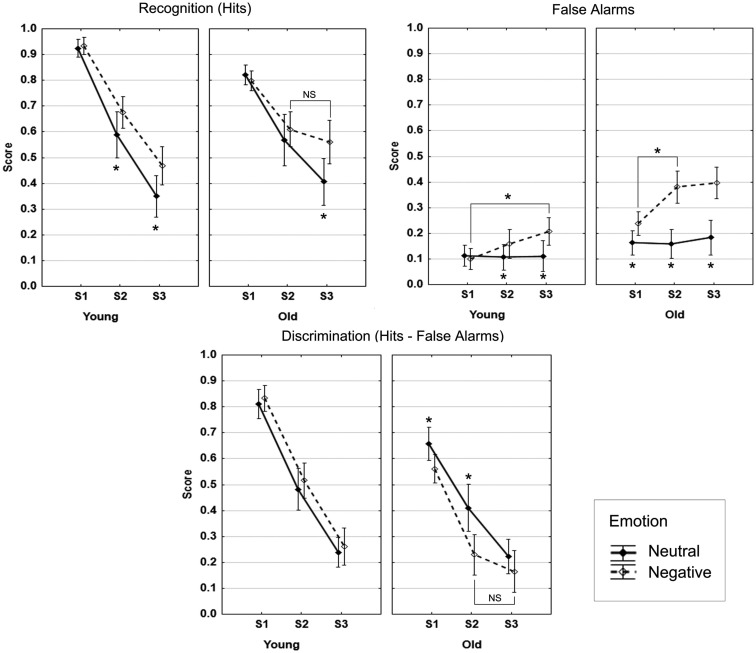
**Recognition data**. Note: the bars denote 95% confidence intervals.

As shown in Figure 2, no main effect of Age was found (*F* < 1). A reduction in hits from S1 through S3 was observed over age groups (*F* = 191.5, *p* < 0.001). An interaction between Age and Session (*F* = 10.0, *p* < 0.001) indicated that at S1 the younger adults produced more hits than the older adults (*F* = 31.3, *p* < 0.001), whereas no difference was found at S2 and S3 (*F* < 1 and *F* = 1.9, *p* = 0.17, respectively). Overall, higher recognition of negative scenes in comparison with neutral scenes was found (*F* = 16.9, *p* < 0.001), and there was no significant interaction between Emotion and Age (*F* < 1). There was a significant interaction effect between Session and Emotion (*F* = 18.9, *p* < 0.001): The negativity effect was significant at S2 and S3 (*F* = 6.2, *p* = 0.02 and *F* = 42.2, *p* < 0.001, respectively), but not at S1 (*F* < 1).

#### False alarms

The older group produced more FA than the younger group (*F* = 22.9, *p* < 0.001), and the overall proportion of FA increased over time (*F* = 8.0, *p* < 0.001), more so between S1 and S2 (*F* = 7.2, *p* = 0.01), the difference between S2 and S3 was not significant (*p* = 0.15). No significant interaction between Age and Session was observed (*F* < 1). More negative than neutral FA were produced (*F* = 71.7, *p* < 0.001). The interaction effect between Age and Emotion (*F* = 23.7, *p* < 0.001) indicated that this effect was more pronounced in the older group (Older: *F* = 79.6, *p* < 0.001; Younger: *F* = 7.3, *p* = 0.01). The significant interaction between Session and Emotion (*F* = 18.9, *p* < 0.001) reflected no increase of neutral FA over the three sessions (*F*s < 1), but a significant increase of negative FA from S1 to S2 (*F* = 15.9, *p* < 0.001; S2–S3: *p* = 0.15). The three-way interaction was not significant (*p* = 0.21). These results are displayed in Figure 2.

#### Discrimination (hits − false alarms)

Taking FA into account had a significant impact on the main age effect and the negativity effect described above for hits (Figure 2). First, overall discrimination was higher in the younger than in the older group (*F* = 22.5, *p* < 0.001). Discrimination decreased over the three sessions (*F* = 243.6, *p* < 0.001), and the interaction between Age and Session (*F* = 6.6, *p* = 0.002) indicated significantly higher discrimination in the younger compared to the older group at S1 and S2 (*F* = 40.6, *p* < 0.001 and *F* = 14.1, *p* < 0.001, respectively), but not at S3 (*F* = 1.9, *p* = 0.17). Discrimination was higher for neutral compared to negative scenes (*F* = 6.6, *p* = 0.002). However, the significant interaction between Age and Emotion (*F* = 17, *p* < 0.001) indicated that the superiority of discrimination of neutral over negative scenes was only significant in the older group (Older: *F* = 20.2, *p* < 0.001; Younger: *F* = 1.3, *p* = 0.26). Also, linear forgetting was seen in the younger group over time for both neutral and negative scenes. Although the three-way interaction was at trend level (*F* = 2.4, *p* = 0.10), we performed follow-up tests that indicated that, in the older group, the discrimination advantage of neutral over negative scenes was significant at S1 and S2 but not at S3 (S1: *F* = 10.4, *p* = 0.003; S2: *F* = 19, *p* < 0.001; S3: *F* = 2.3, *p* = 0.14). Moreover, higher discrimination of neutral scenes in the younger compared to the older group was significant only at S1 (*F* = 13.9, *p* < 0.001), and discrimination was significantly higher in the younger group for negative scenes at S1 and S2 (*F* = 55.7, *p* < 0.001 and *F* = 31.9, *p* < 0.001, respectively), but only at trend level at S3 (*F* = 3.3, *p* = 0.08).

Over the three ANOVAs, assessment of power of the significant effects was performed, as the sample size was quite small. Considering 0.80 as the cut-off, all significant main effects and interactions were above this threshold (0.90 < power < 1) except the main effect of emotion for discrimination (neutral > negative) where the power was of 0.71.

In summary, taking into account false recognitions, which greatly increased over time for negative scenes, canceled out the negativity effect that was seen for hits in both the younger and older groups, this effect being more pronounced in the old. Contrary to our prediction, no negativity effect was observed for discrimination in the two age groups.

### Neuroimaging results

#### Successful long-term retrieval by age group

##### Hits vs. misses

**Figure 3:**
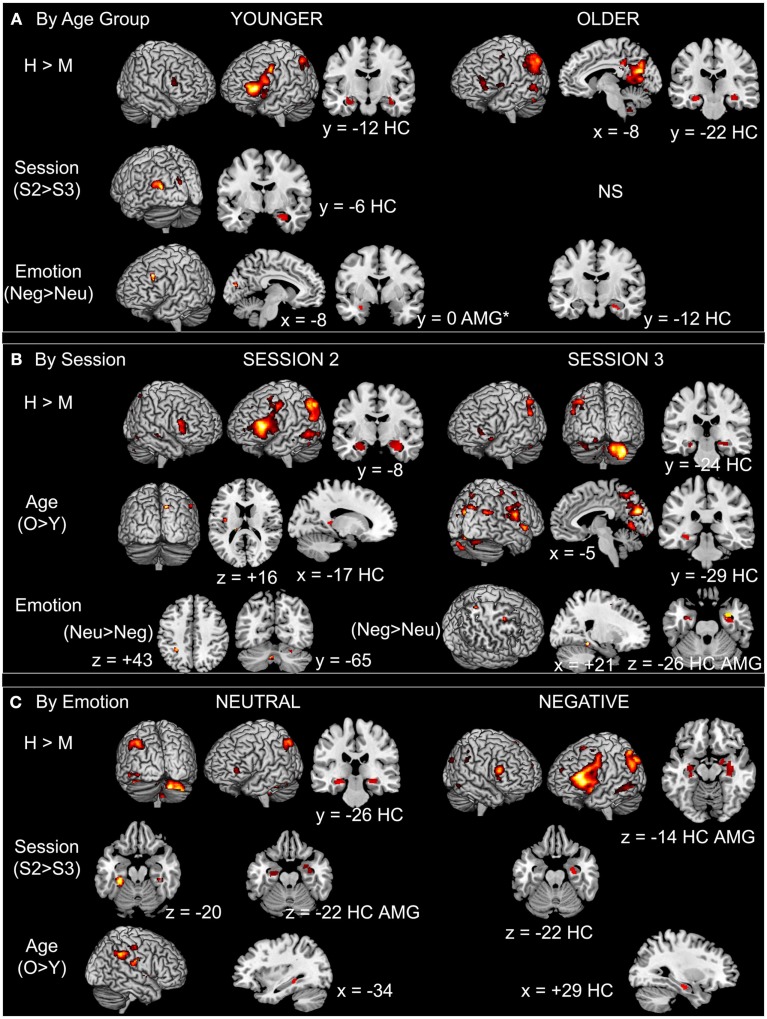
**Main effects in BOLD activation patterns by (A) age group, (B) session, and (C) emotion**. Notes: AMG, amygdala; H, hits; HC, hippocampus; M, misses; Neg, negative; Neu, neutral; O, older; S2, Session 2; S3, Session 3; Y, younger. Whole-brain results shown at *p* < 0.001 uncorrected, and ROI results (HC, AMG) shown at *p* < 0.0125. *In the younger group the cluster size of the emotion effect in the AMG is below the extent threshold set for significance (*k* = 13 < 20 voxels).

As shown in Figure 3A (see also Table A2 in Appendix), in the younger group, successful recognition was characterized by strong left PFC activity (ventro- and dorso-lateral) as well as activity in left lateral parietal cortex and right cerebellum. Significant activity linked to successful recognition in the young was also detected in bilateral HC and AMG. In the older group, the strongest activity for this contrast was located in posterior areas such as precuneus, posterior cingulate, and retrosplenial cortex, lateral parietal and occipito-temporal areas, as well as cerebellum. A smaller cluster located in left ventrolateral PFC (VLPFC) also showed significant activity. Bilateral HC was also significantly more activated for H than for M.

##### Session

In the younger group, the strongest session difference was found in occipital areas and striatum, with more activity at S2 than at S3. HC and right AMG were also more activated at S2 in comparison with S3 in the young (Figure 3A; Table A2 in Appendix). In the older group, no effect of session was found.

##### Emotion

In the younger group, left lateral PFC was more activated in the negative condition in comparison with the neutral condition; we also found activity in left AMG but below the chosen extent threshold (*p* < 0.0125, but *k* = 13 voxels). In the older group, only an area in right HC was more activated during successful recognition of negative scenes in comparison with neutral scenes. In no case did successful recognition of neutral scenes elicit more activity than negative scenes (Figure 3A; Table A2 in Appendix).

##### Session × emotion interaction

**Figure 4:**
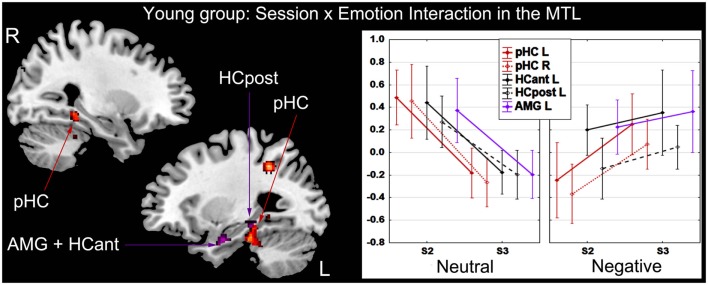
**Interaction effect between session and emotion in the younger group**. There was a significant decrease of activity during successful recognition of neutral scenes from S2 to S3 in the MTL, a significant increase of activity in posterior parahippocampal cortex bilaterally and stable activity over time in left hippocampus and amygdala during successful recognition of negative scenes. The *Y*-axis of the graph represents contrast values (Hits − Misses). L, left; R, right; HCant, anterior hippocampus; HCpost, posterior hippocampus; pHC, parahippocampal cortex; AMG, amygdala; MTL, medial temporal lobe; S2, Session 2 (1-week delay); S3, Session 3 (3-week delay).

In the younger group, activity of several regions mainly located in medial temporal lobe (MTL) varied as a function of session and emotion. At the whole-brain level, activity in posterior parahippocampal cortex (bilaterally) and left lateral parietal cortex diminished from S2 to S3 for successful retrieval of neutral scenes, whereas activity in these regions increased for successful retrieval of negative scenes. At the ROI level, activity in left HC and AMG diminished from S2 to S3 for successful retrieval of neutral scenes, whereas it remained stable for successful retrieval of negative scenes (Figure 4; Table 2). In the older group, the Session × Emotion interaction was not significant for any brain region.

**Table 2:** **Interaction between retrieval session and emotion in the younger group**.

Brain areas	BA	MNI coordinates			*t*	*k*	*F*-tests	
		*x*	*y*	*z*			Neutral (S2 > S3)	Negative (S3 > S2)
ROI HC L		−32	−32	−12	3.65	63	*p* = 0.003	NS
		−30	−6	−28	2.93	36	*p* = 0.002	NS
ROI AMG L		−28	−4	−24	2.80	23	*p* = 0.004	NS
ParaHC, fusiform L	37, 30	−32	−32	−16	4.16	220	*p* = 0.0004	*p* = 0.008
ParaHC, fusiform R	37, 30	22	−40	−12	4.10	190	*p* = 0.0001	*p* = 0.01
Parietal inferior L	40	−28	−46	42	4.09	82	*p* = 0.01	*p* = 0.002
Parietal superior L	7	−22	−72	48	3.43	64	*p* = 0.0001	NS
DMPFC L	6	−22	0	52	3.67	35	*p* = 0.02	*p* = 0.01
Postcentral R	3	58	−16	44	3.61	26	*p* = 0.0002	NS

*All results shown at *p* < 0.001 except ROI analyses at *p* < 0.0125 for HC (first cluster: peak at *p* < 0.001; second peak at *p* = 0.002) and AMG (peak at *p* = 0.003). AMG, amygdala; BA, Brodmann Area; DMPFC, dorsomedial prefrontal cortex; HC, hippocampus; L, left; MNI, Montreal Neurological Institute; NS, no significant; ParaHC, parahippocampal cortex; R, right; ROI, region of interest; S2, Session 2 (1-week retention interval); S3, Session 3 (3-week retention interval)*.

#### Successful long-term retrieval by session

##### Hits vs. misses

As shown in Figure 3B (see also Table A3 in Appendix), activity was found at S2 in left PFC as well as in posterior cortical areas, and also in the HC bilaterally and the right AMG. By contrast, more cerebellar activity was seen at S3, where more HC activity was also revealed.

##### Age

The older adults showed more activity in comparison with their younger counterparts at both sessions and mostly in posterior brain areas; this effect was most pronounced at S3 (Figure 3B; Table A3 in Appendix).

##### Emotion

At S3, successful retrieval of negative scenes elicited more activity in HC bilaterally and in right AMG in comparison with retrieval of neutral scenes. Activity in right parahippocampal/fusiform cortex, dorsomedial PFC, and precuneus showed the same pattern. By contrast, at S2, other regions (cerebellum and inferior parietal cortex) showed more activity during retrieval of neutral in comparison with negative scenes (Figure 3B; Table A3 in Appendix).

##### Age × emotion interaction

**Figure 5:**
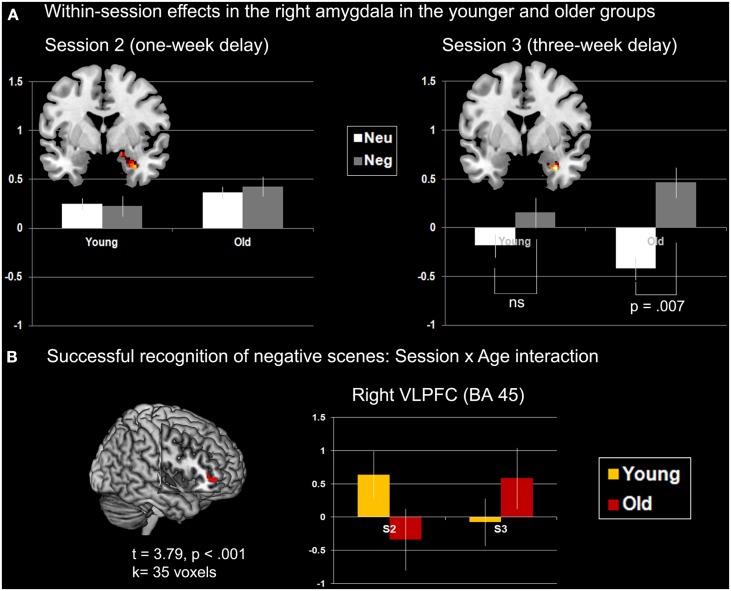
**Interaction effects with age in AMG and VLPFC**. **(A)** Within-session effects in right AMG (coronal slices, *y* = 0). The main effect of successful retrieval (H > M) at S2 is plotted on the left. No modulation of AMG activity was found according to Age and Emotion. The main effect of Emotion (negative > neutral) at S3 in plotted on the right. Modulation of AMG activity differed across Age: in the younger group the effect of emotion was not significant (*p* = 0.23), although it was reliable in the older group (*p* = 0.007). The *Y*-axis of the graphs represents contrast values (H > M) which have been extracted individually over all voxels of the cluster (eigenvalues). Bars represent standard errors. **(B)** Age × Session interaction for successfully recognized negative scenes. The *Y*-axis of the graph represents contrast values (H > M). Peak coordinates are [46; 46; 0].

No significant effects were found at S2 and S3. Nonetheless, plotting the effect of Emotion (Negative > Neutral) described above in right AMG (Figure 5A) suggested the existence of an interaction effect, such that the main effect of Emotion was driven by the older group. Follow-up analyses confirmed that at S3 the difference of AMG activity between neutral and negative scenes was significant in the older group only, at *p* = 0.007, with more activity for negative scenes.

#### Successful long-term retrieval by emotional valence

##### Hits vs. misses

As shown in Figure 3C (and Table A4 in Appendix), for both the neutral and negative scenes, HC was activated bilaterally, whereas AMG was activated only for negative scenes, especially in the right hemisphere (subthreshold in the left, *k* = 13 voxels). Although activity was found in similar regions for both types of scenes such as VLPFC and parietal areas with a strong asymmetry toward the left hemisphere, more widespread activity was found for the negative scenes where other areas were also activated (cingulate gyrus, occipital and temporal regions, and thalamus).

##### Session

At the whole-brain level, only a small region in right cerebellum showed increased activity at S3 in comparison with S2 for the negative scenes. Further, activity in HC dropped from S2 to S3 for both types of scenes, but the effect was much more pronounced for the neutral scenes: Although only a small portion of right HC showed decreased activity for negative scenes, larger HC areas bilaterally showed such a decrease for neutral scenes. Moreover, although AMG was not activated for the main effect (H − M), we found decreased activity over time in right AMG for the neutral (but not the negative) scenes (Figure 3C; Table A4 in Appendix).

##### Age

For both types of scenes, older adults showed more activity in comparison with younger adults. This effect was seen in posterior brain regions (occipital, parietal, and lateral temporal regions) and to a lesser extent in HC (Figure 3C; Table A4 in Appendix).

##### Session × age interaction

No brain area showed a significant interaction for the neutral scenes. For negative scenes, activity of right VLPFC (BA 45) increased from S2 to S3 in the older group, whereas it decreased in the younger group over time (Figure 5B; Table A4 in Appendix). To verify whether this effect was truly unilateral, we used a more permissive threshold, *p* < 0.005. Left VLPFC became significant ([−36; 36; 6], *t* = 3.19), likely suggesting bilateral VLPFC activity modulation according to session and age. Bilateral involvement may reflect complementary processes according to the nature of the material (visual and verbal).

#### Correlational analyses

Correlations were performed between activity in AMG, VLPFC, and HC, and between activity in these areas and performance for negative scenes, both Hits and Discrimination. Four older individuals were considered as outliers in these analyses as their brain data were >2 SDs and were therefore excluded from these analyses. Results are shown in Table 3, but should be considered cautiously due to the small sample size.

**Table 3:** **Within-group and within-session correlations between brain activity and performance**.

	Younger		Older	
	S2	S3	S2	S3
**AMG R**				
VLPFC R	0.49*	−0.53*	−0.30	−0.13
VLPFC L	0.68**	−0.16	0.32	−0.02
HC R	0.56**	0.41	0.26	0.15
HC L	0.49*	0.36	0.03	−0.26
Neg hits	0.54*	0.62**	−0.16	0.18
Neg disc	0.53*	0.40	−0.32	0.43
**VLPFC R**				
HC R	0.46*	−0.25	0.64*	0.33
HC L	0.02	−0.28	0.42	0.24
Neg hits	−0.03	−0.60**	0.67**	−0.01
Neg disc	0.08	−0.23	0.37	0.02
**VLPFC L**				
HC R	0.59**	−0.05	0.37	0.17
HC L	0.71**	−0.05	0.40	0.22
Neg hits	0.40	−0.42	0.35	−0.09
Neg disc	0.53*	−0.23	0.41	0.29
VLPFC R – VLPFC L	0.52*	0.66**	0.41	0.31
**HC R**				
Neg hits	0.24	0.14	0.37	−0.40
Neg disc	0.35	0.15	0.28	−0.33
**HC L**				
Neg hits	0.61**	0.25	0.20	−0.26
Neg disc	0.60**	0.22	0.14	−0.34
HC R–HC L	0.53*	0.58**	0.68**	0.65**

*p ≤ 0.05, **p ≤ 0.01. Younger: *N* = 19. Older Session 2: *N* = 14 for correlations involving AMG R, *N* = 13 for correlations involving HC, otherwise *N* = 15. Older Session 3: *N* = 14 for correlations involving AMG R, *N* = 14 for correlations involving VLPFC, *N* = 13 for the correlations between AMG R and VLPFC, otherwise *N* = 15. AMG, amygdala; HC, hippocampus; L, left; Neg Disc, negative discrimination; R, right; S2, Session 2 (1-week delay); S3, Session 3 (3-week delay); VLPFC, ventrolateral prefrontal cortex.

In the younger group at S2, positive correlations were found between activity in AMG and VLPFC. As VLPFC was significantly activated at the group level, the significant correlations with AMG activity indicate that these two regions may interact during successful recognition of negative scenes. Correlations with performance mostly indicate that left- but not right-sided structures (HC, VLPFC) contributed to performance, except for right AMG whose activity was positively linked to performance. However, the fact that right HC and VLPFC were significantly correlated with AMG suggests an indirect contribution to performance. At S3, activity in AMG was positively correlated with performance (negative hits), but in contrast with S2, right (but not left) VLPFC activity was negatively correlated with the proportion of negative hits. However, at this session, VLPFC was not significantly activated during retrieval of negative scenes at the group level. Therefore, it can be hypothesized that mainly automatic mechanisms as subserved by the AMG (as opposed to controlled processes subserved by the PFC) are involved in successful recognition of negative scenes in younger adults. The opposite role of these two regions was substantiated with the negative correlation between activity in AMG and right VLPFC. In contrast with S2, HC activity did not correlate with any of the other measures at S3, suggesting diminished contribution of this structure to performance with increasing retention interval.

In the older group, at S2, activity in right AMG did not correlate with performance, whereas right (but not left) VLPFC activity correlated positively with negative hits, suggestive of beneficial controlled mechanisms, in interaction with right HC, whose activity was also correlated with right VLPFC. However, right VLPFC was not significantly activated at the group level during successful retrieval of negative scenes, which goes against a compensatory interpretation. VLPFC was activated only at S3, suggesting delayed involvement of this region compared with the younger group. However, the lack of correlation with AMG indicates that these two regions may not interact in older adults. Actually, at S3, no correlation was significant in the older group, although there was a trend toward a positive relationship between AMG activity and discrimination of negative scenes (*r* = 0.43, *p* = 0.13). If this trend is meaningful, one can hypothesize that successful retrieval of negative scenes at S3 is mainly driven by automatic processes subserved by AMG activity in older adults.

Finally, while in the younger group activity between left- and right-sided structures was significantly correlated (HC, VLPFC), only activity between left and right HC was correlated in the older group, suggestive of a functional frontal disconnection in aging.

Overall, AMG activity was positively correlated with performance in the younger group in the two retrieval sessions, suggesting a specific role of the AMG in successful recognition of negative scenes regardless of time, and a trend toward a similar relationship in the older group was only seen after 3 weeks retention. Interestingly, at Session 2, only activity in left VLPFC and HC was associated with performance in the young group. Regarding activation of left and right VLPFC in the older group at S3, no relationship was found with either AMG activity or performance, suggesting no compensation or increased emotional regulation at retrieval.

#### Successful, false, and true recognition of negative scenes in the older group

**Figure 6:**
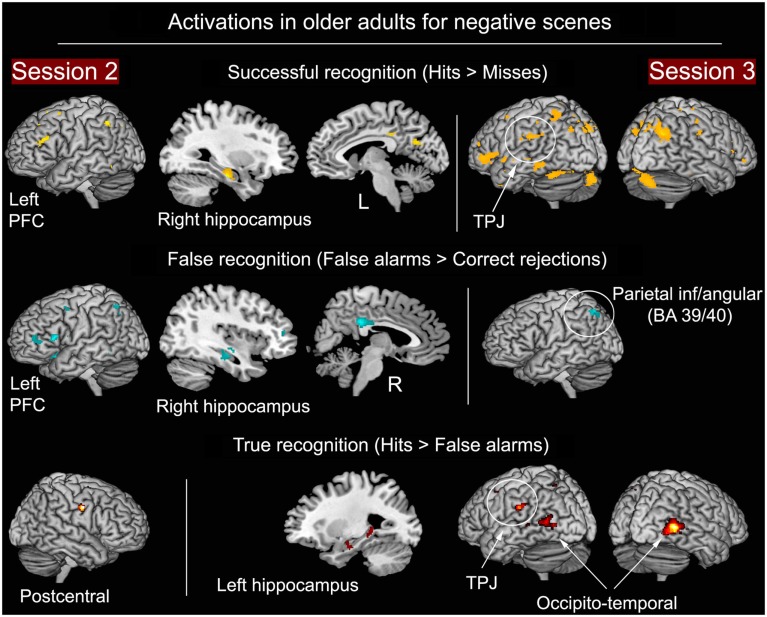
**Successful, false, and true recognition of negative scenes in the older group at S2 and S3**. Notes: all results shown at *p* < 0.001 (whole-brain analyses), except HC (*p* < 0.0125, ROI analyses).

As shown in Figure 6 and Table 4, at S2, same areas were activated during successful (H > M) and false (FA > CR) recognition, including right HC and left fronto-parietal regions. Consequently, the direct comparison between H and FA showed very few differences, suggestive of similar neurocognitive processes involved in successful and false retrieval. At S3 the pattern of results was different. More activity in several regions was found during successful in comparison with false recognition. The direct contrast between H and FA, thought to unveil brain areas associated with true recognition, showed more activity for H in lateral occipito-temporal areas, left temporo-parietal junction, and left HC.

**Table 4:** **Brain activity during successful (H > M), false (FA > CR), and true (H > FA) recognition of negative scenes in older adults at Session 2 and Session 3**.

Brain areas	BA	MNI coordinates			*t*	*k*	*p* (ROI-peak)
		*x*	*y*	*z*	
**Session 2: Neg H > Neg M**							
HC R ROI		28	−16	−22	3.98	91	0.001
Parietal inferior L	40	−42	−50	46	5.10	37	
VLPFC L	45	−44	32	24	4.76	53	
Cingulate middle/posterior L	23	−6	−32	34	4.52	37	
Precuneus/cuneus L	23	−6	−64	24	4.52	38	
**Session 3: Neg H > Neg M**							
Occipital middle/superior, precuneus LR	19, 7	34	−70	34	11.45	1730	
Anteromedial PFC/cingulate anterior L	32, 10	−2	52	14	8.03	186	
Cingulate anterior L	32	−10	34	18	6.69	157	
Cerebellum crus 1, 2 R		24	−82	−38	6.59	908	
Rostromedial PFC R	10	4	56	10	6.11	28	
VLPFC, insula L	45, 47	−48	34	0	5.81	354	
Cingulate middle LR	23	6	−10	28	5.74	77	
Temporo-parietal junction L	22, 40	−54	−42	28	5.67	79	
VLPFC R	45	42	28	24	5.51	25	
Parietal inferior R	40	32	−46	34	5.44	37	
Rostrolateral PFC L	10	−26	58	14	5.36	46	
Precuneus L	5	−10	−52	68	5.18	56	
Lingual L	18	−14	−52	0	5.13	38	
Rostrolateral PFC L	10	20	64	8	5.08	26	
Temporal inferior L	37	−56	−54	−10	5.01	81	
Temporal superior L	22	−56	−2	−4	5.00	25	
Precuneus R	5	4	−46	60	4.98	24	
Parietal superior L	7	−30	−60	60	4.85	96	
Superior frontal sulcus L	6	−32	6	52	4.66	32	
Cingulate posterior LR	26	0	−30	24	4.64	122	
VLPFC L	45	−44	44	18	4.63	23	
Postcentral R	3	32	−30	56	4.58	24	
VLPFC L	47	−36	26	28	4.44	22	
Lingual L	17	−6	−70	6	4.41	36	
**Session 2: Neg FA > Neg CR**							
HC R ROI		36	−16	−14	5.34	175	<0.001
Cingulate middle/posterior LR	23	2	−34	30	6.78	278	
VLPFC L	45	−52	24	10	6.74	71	
Precuneus L	23	−10	−64	30	6.13	49	
Insula, HC R		40	−12	−12	5.6	92	
VLPFC L	47	−36	20	−16	5.34	33	
VLPFC R	45	50	24	8	5.09	25	
Parietal inferior L	40	−46	−56	54	4.95	28	
VLPFC L	47	−38	40	4	4.52	56	
**Session 3: Neg FA > Neg CR**							
Parietal inferior/angular L	39/40	−42	−62	54	7.03	51	
**Session 2: Neg H > Neg FA**							
Postcentral R	4	56	−2	36	4.98	44	
**Session 2: Neg FA > Neg H**							
Insula R		40	−12	−12	4.64	20	
**Session 3: Neg H > Neg FA**							
HC L ROI		−24	−40	4	5.77	70	<0.001
		−30	−8	−16	3.39	36	0.002
AMG L ROI		−28	−8	−12	2.72	1	0.008*
Occipito-temporal R	37, 19	52	−66	6	6.05	408	
Lingual L	18	−8	−60	−4	5.42	45	
Occipito-temporal L	37, 19	−48	−84	4	5.37	116	
Temporo-parietal junction L	48	−56	−38	26	5.14	86	
Calcarine, precuneus L	17	−4	−68	10	4.78	27	
**Session 3: Neg FA > Neg H**							
	–						

*At *p* < 0.05: AMG L 15 voxels; AMG R [32; 2; −22], *t* = 2.26, *p* (peak) = 0.02, *k* = 15 voxels. AMG, amygdala; BA, Brodmann Area; CR, correct rejections; FA, false alarms; H, hits; HC, hippocampus; L, left; MNI, Montreal Neurological Institute; Neg, negative; R, right; ROI, region of interest; VLPFC, ventrolateral prefrontal cortex.

#### HC and AMG age-related atrophy

Because age-related differences were observed in the fMRI data for HC and AMG, we examined whether these effects could be due to local atrophy. There were two reasons for these additional analyses: first, several recent studies have demonstrated that more, or less, brain activity in older adults was partly driven by gray-matter losses, thus providing a biological underpinning for activity difference in aging (e.g., Kalpouzos et al., 2012; for review, see Kalpouzos and Nyberg, 2012); second, as noted in the introduction, a previous study on emotional memory in aging (St Jacques et al., 2009b) showed an hemispheric asymmetry in AMG activity in younger and older adults (more left activity in the young and more right activity in the old) that is difficult to explain. The current fMRI results showed: (1) More HC activity in older compared to younger adults, (2) Bilateral AMG involvement in the young, but mostly right AMG activity in the old (as in the St Jacques et al.’s study). After transformation of the functional HC and AMG clusters into ROIs, we overlaid the newly created binary masks on the individual unsmoothed, MNI-normalized and modulated gray-matter images preprocessed with the VBM8 toolbox as mentioned in the Section “Materials and Methods,” and extracted with MRIcroN the mean intensity for each cluster of interest, representing GM volume in this specific area^2^. We found no significant atrophy in the investigated HC areas that showed more activity in the older compared to the younger group [Clusters: (1) Age effect in Session 2: *t* = 0.69, *p* = 0.49; (2) Age effect in Session 3: *t* = 1.73, *p* = 0.09; (3) Age effect in Negative condition: *t* = 1.73, *p* = 0.09; and (4) Age effect in Neutral condition: *t* = 1.74, *p* = 0.09]. Similarly, we found no evidence for atrophy in right AMG, but left AMG showed significant gray-matter volume loss in the older group [Clusters for left AMG: (1) Emotion × Session interaction in the younger group: *t* = 2.2, *p* = 0.03; (2) Negative − Neutral in the younger group: *t* = 3.45, *p* = 0.001; and (3) H − M in the Negative condition: *t* = 2.04, *p* = 0.05. Clusters for right AMG: (1) Negative − Neutral for Session 3: *t* = 0.96, *p* = 0.34; (2) H − M in Negative condition: *t* = 1.94, *p* = 0.06; and (3) Session 2 − Session 3 in Neutral condition: *t* = 0.95, *p* = 0.35]. These findings suggest that the age-related HC over-activation is unlikely to be driven by gray-matter losses, and that the lateralization effect seen in AMG may be due to structural deterioration of left AMG in aging. Specifically, in this task context only the structurally intact right AMG may be used to perform the emotional recognition task in older adults.

## Discussion

The main aims of this study were to test the negativity effect over 1 and 3 weeks in younger and older adults, and to investigate the brain correlates of long-term successful, false, and true recognition. In long-term recognition, the negativity effect found for hits was canceled out when taking into account false recognitions, and this effect was present in both groups with a magnification of the effect in the older adults. MTL activity, including HC and AMG, was present in general during successful recognition of negative scenes, but its activity was modulated according to session of retrieval and age. Findings related to brain regions involved during successful and false recognition of negative scenes in older adults are discussed in terms of impaired recollection processes.

### Testing the negativity effect

Although previous studies showed a robust negativity effect in young adults such that negative items are better remembered than neutral items, findings are mixed in older adults, with a trend toward a reduced negativity effect in true recognition. However, in most of these studies, the delay of retention was short (for meta-analysis, see Murphy and Isaacowitz, 2008). Here we show that, for hit rates, where no age-related difference was found in performance, both younger and older adults showed a negativity effect that increased over time. Further, more false recognitions of negative scenes were observed over time; this effect was especially marked in the old. Thus, H and FA canceled each other out, resulting in an absence of a negativity effect in the younger group in terms of discrimination, and higher discrimination of neutral than negative scenes in the older group over the three retrieval sessions. Also, global discrimination was higher in the younger group, except after 3 weeks of retention where age differences no longer were observed.

These results are in accordance with the view that negative emotional stimuli are more prone to memory distorsion than neutral stimuli. This may reflect that emotional items generally have a higher semantic cohesiveness; they share more characteristics within categories than neutral items (Maratos et al., 2000; Marchewka et al., 2008). Indeed, contrary to the neutral scenes, the negative scenes in the IAPS can be grouped into categories like “snakes,” “guns,” and “blood” (Figure 1). In line with our hypothesis, the findings suggest that older adults, relatively to younger adults, are more prone to falsely recognize a negative scene because they may base their recognition on categories or more general features rather than on perceptual detail.

Second, our results extend Howe et al.’s (2010) findings on young adults to older adults, showing that the increase of false emotional memories over time is magnified in aging. This disproportionate increase of FA in the older group was striking; the percentage of negative FA was 40% at both S2 and S3. Although the results suggest stabilization of performance from week 1 to 3 with no further forgetting of negative scenes, no further increasing proportion of false negative recognitions in the older adults, and normalization of performance in comparison with the younger group over the last 2 weeks, these effects were likely a consequence of a floor effect. Indeed, older adults displayed a rapid decline of performance already at Session 2, reaching a very low discrimination score (but not proportion of hits) at Session 3. Overall, memory accuracy for negative scenes was not better than that of neutral scenes, especially in older adults, thus supporting Socioemotional selectivity theory in aging (Mather, 2012).

However, a recent study from our lab with a retention interval of 1 year demonstrated better memory accuracy for negative compared to neutral scenes in both younger and older adults (Gavazzeni et al., 2012). This further highlights the importance of retention intervals and might reflect that very long-term consolidation of negative scenes may result in a re-established negativity effect in both age groups. Perhaps more importantly, the lack of negativity effect in the younger group on long-term memory accuracy may be due to the material used. As aforementioned, IAPS contains many items that can easily be grouped into semantic categories. Information regarding this possible grouping of items into categories is usually not provided in previous studies, and differences on this dimension across studies may explain discrepant findings. Indeed, in their study on false memory, Howe et al. (2010) showed, in young adults, no valence effects for unrelated lures on FA rates, but significant higher FA rates for related negative emotional lures than for related neutral lures, substantiating the importance to consider this factor in emotional memory studies.

### Emotion and session of retrieval modulate MTL activity

The involvement of HC has been the focus of studies in which retention interval has been manipulated (Andreasen et al., 1995; Stark and Squire, 2000; Dupont et al., 2001; Bosshardt et al., 2005a,b; Takashima et al., 2006, 2009; Janzen et al., 2008; Suchan et al., 2008; Viskontas et al., 2009). A controversy exists in the literature, with two opposing theories: Multiple trace theory claims that HC is systematically engaged when retrieving information from long-term memory whatever the remoteness, whereas the standard model posits that HC disengages with increasing remoteness (for review, see Winocur and Moscovitch, 2011). Research has largely ignored the role of emotion in modulating the effect of passage of time on HC activity. Our findings underscore the point that emotion modulates the engagement of the MTL during recognition of scenes over time, but only in young adults (Figure 4). For HC and other MTL structures (posterior parahippocampal cortex bilaterally and left AMG), activity decreased over time for successful recognition of neutral scenes, in agreement with the standard model. However, activity remained stable or increased for successful recognition of negative scenes, in line with Multiple trace theory. Additional correlations between HC activity and performance during successful recognition of negative scenes revealed that increased activity in left HC was associated with higher performance in young adults after 1 week but not after 3 weeks of retention, indicative of reduced function of the structure at the cognitive level with increasing remoteness.

### Aging effects on the neural substrates of long-term successful retrieval

Both the younger and older groups showed activity in brain areas typically associated with successful episodic retrieval such as fronto-parietal areas and HC (Figure 3). This pattern of activity was strongly left-lateralized regarding neocortical regions. Retrieval in episodic memory has been traditionally related to right-sided activity notably in the PFC (Tulving et al., 1994); however a recent meta-analysis showed that *successful* retrieval elicited more left-lateralized regions (Spaniol et al., 2009). The left-sided preference found here could also reflect task difficulty. Some previous studies showed a hemispheric modulation according to the difficulty of the task and the specific cognitive processes needed to solve such tasks (Nolde et al., 1998; Cabeza et al., 2003). Although we used a recognition task, supposedly easy to carry out, the 1- and 3-week delays of retention may have contributed to increased task difficulty, where subjects may have had to generate more cues in order to decide whether a scene had been seen or not 1 and 3 weeks ago.

#### Overall age-related effects

Generally, more BOLD activity was found in the older compared with the younger group. This pattern varied by session and emotion. The most pronounced age difference was seen for Session 3 in posterior areas, notably the precuneus, which is known to be involved in imagery processes and episodic retrieval (Huijbers et al., 2011). Two invariant patterns were observed across the different conditions: Older individuals systematically activated HC more than the young, whereas PFC over- or under-activation in aging was generally not evidenced. The latter result goes against many studies on age-related differences in functional brain activity, particularly in episodic memory, often showing more recruitment of PFC in aging (Rajah and D’Esposito, 2005). This may be due to the fact that, unlike most previous assessments, the retrieval sessions here were delayed, making the task more difficult, even for younger subjects, as substantiated by the recruitment of left PFC in the main effect of successful retrieval (Figure 3A). More HC activity is a rare finding in normal aging, although it has been shown in persons with mild cognitive impairment (Putcha et al., 2011): Over-activation, in that case, was seen as compensatory for structural MTL deterioration. As no evident hippocampal atrophy was found, the over-recruitment of the old found here may be due to the length of the retention interval and accompanying increase in difficulty. One study showed that increased cognitive demands during an associative encoding task increased both PFC and HC activity similarly in younger and older adults (Leshikar et al., 2010), corroborating the hypothesis that task difficulty, due to the length of retention interval, resulted in minimal age differences in PFC activity and slightly increased HC activity in the older group.

#### Right amygdala activity in older adults after 3 weeks of retention for successful recognition of negative scenes

While AMG engagement during perception and encoding of negative stimuli is well established (Murty et al., 2010; Sabatinelli et al., 2011), its involvement during emotional episodic retrieval is not obvious, and even less so during *successful* retrieval. The present study, in which an event-related fMRI design was used, showed that over the two groups, right AMG activity was present for successful retrieval of negative scenes but not for neutral scenes (Figure 3C). Within-group analyses further showed that left AMG displayed more activity during successful recognition of negative in comparison with neutral scenes in younger adults (Figure 3A), with a modulation effect according to session (Figure 4), whereas no significant effect of emotion on AMG activity could be demonstrated in older adults.

When considering the retrieval sessions separately over the two groups, an effect of negative emotion was revealed after 3 weeks of retention in right AMG, but not after 1 week (Figure 3B). Thus, AMG activity was specifically involved in successful long-term retrieval of negative scenes (Dolcos et al., 2005). Examining the effect of emotion for Session 3 further revealed that this effect was mainly driven by the older adults, who showed a significant difference for this session between successful negative and neutral scene recognition (Figure 5A). Specifically, after a long delay, right AMG was especially activated during successful retrieval of negative scenes in older individuals, suggesting that automatic mechanisms, as mediated by AMG, may be activated after a certain time in older adults during successful recognition of negative information. Although the positive correlation observed in this group between AMG activity and discrimination of negative scenes was only at trend level, significant positive associations found in the younger group support the hypothesis of AMG involvement in recognition memory of negative scenes after 1 and 3 weeks of retention. For the older group, however, it is difficult to state that increased right AMG activity for successfully recognized negative scenes compared with neutral scenes subtended a delayed negativity effect: Considering the discrimination findings, although performance seemed to stabilize compared to S2 in the old, and normalize compared to the younger group, the fact that performance was low prevents firm conclusions. However, a beneficial effect of AMG activity on performance can be hypothesized when considering hits only, where the negativity effect appeared at S3 in the older group (see also Gavazzeni et al., 2012).

#### VLPFC involvement during successful recognition of negative scenes

An interaction between Age and Session for successful recognition of negative scenes was observed in right VLPFC (and left VLPFC at a more liberal threshold): activity in this region decreased from S2 to S3 in the younger group, while it increased in the older group. In other words, VLPFC was engaged in the younger, but not older adults at S2, while it was engaged at S3 in the older but not in the younger group (Figure 5B). Right VLPFC has been shown to play a role in response inhibition and selective attention (Aron et al., 2003, 2004), especially in relation to episodic memory retrieval (Kuhl et al., 2007, 2008; for review see Anderson and Weaver, 2009), where this region is thought to select the correct representation among several plausible alternatives (by inhibiting irrelevant options). Given the nature of the task (yes-no recognition with targets and lures), the latter function seems plausible. In the younger group, left and right VLPFC were activated at S2 (not at S3), and activity in these regions was positively correlated with AMG activity, but only left VLPFC was correlated with performance. This suggests a direct contribution of left VLPFC to performance, and an indirect beneficial effect of right VLPFC on performance, via AMG, whose activity was positively linked to successful recognition of negative scenes (Table 3). The same conclusion can be drawn for HC: at S2, only left HC was correlated with performance, suggesting a direct contribution to performance, and the significant correlations between right HC and AMG (as well as left and right VLPFC) may indicate an indirect contribution of these right-lateralized structures on performance via AMG activity. If we consider theoretical models on hemispheric asymmetries, left structures being more involved in episodic and/or verbal processes and right structures being more engaged in visual processes (Kalpouzos and Nyberg, 2010), our findings suggest a direct contribution of episodic/verbal processes subserved by left VLPFC and HC on successful recognition, and indirect contribution of the contralateral structures subserving visual mechanisms via the right AMG, which directly contributed to successful recognition of negative scenes. Also, and as aforementioned in Section “Overall Age-Related Effects,” increased task difficulty due to long retention interval may have been a factor of the involvement of left brain areas, directly influencing performance via additional episodic, verbal-related processes.

In the older group, activity in VLPFC was delayed in comparison with the younger group, as activity was seen only at S3. Nonetheless, at S3, no significant correlation was seen between VLPFC and AMG activity, and performance did not correlate with VLPFC activity in the old. Hence, increased activity in VLPFC in the older group did not seem to play a role during successful recognition of negative scenes, and may therefore reflect a failed attempt of either compensation or emotion regulation, which is in disagreement with previous studies where PFC activity in older adults was interpreted as compensatory (Murty et al., 2009) or reflecting increased regulation of emotional processes (St Jacques et al., 2009a). This does not exclude the hypothesis of increased regulation of emotion at encoding (Mather, 2012). Nevertheless, the absence of correlation between AMG and VLPFC activity in the old is partly in line with St Jacques et al.’s (2009b) functional connectivity findings, where a decrease in functional connectivity between AMG and VLPFC was found in older compared with younger adults, but an increase in functional connectivity between AMG and dorsolateral PFC. These differences highlight the need to consider age effects on different regions of the PFC (see also Ebner et al., 2012 for an investigation of a differential involvement of dorso- and ventro-medial PFC in emotion in younger and older adults; for review, see Mather, 2012).

Interestingly, interhemispheric correlations suggest a preserved age-related functional connectivity between left and right HC, but a functional disconnection between contralateral VLPFC areas, the latter indicating reduced interhemispheric information flow between these anterior regions. Anterior callosal integrity has been shown to be crucial for interhemispheric functional connectivity of the frontal lobes (Davis et al., 2012). Age-related structural deterioration of the corpus callosum may contribute to the observed diminished relationship between activity in left and right VLPFC as observed in the present study. Future studies combining fMRI and Diffusion Tensor Imaging in emotional pictorial memory could address this hypothesis.

### Brain correlates of successful, false, and true recognition of negative scenes in older adults

Similar regions were engaged during successful recognition (as assessed using the H − M contrast) and false recognition (FA − CR), including left VLPFC, middle-posterior cingulate and HC at S2, and left lateral parietal cortex at S2 and S3 (Figure 6). Left VLPFC has been associated with retrieval of semantic information (Tulving et al., 1994), contributing to episodic memory retrieval (see also Aging Effects on the Neural Substrates of Long-Term Successful Retrieval of the present discussion). The fact that the direct comparison between hits and FA did not show differential activity in this region suggests that, in both successful and false recognition, left VLPFC is linked to semantic operations during episodic retrieval by providing categorical or general information, but does not, in the present case, contribute to true recognition. Failure to find significant correlations between left VLPFC activity and accurate recognition in the old group partly substantiate this interpretation (Table 3).

Brain areas differentially activated for successful and false recognitions were mostly seen at S3. Lateral occipito-temporal cortex was more activated for H than for FA for negative scenes in the older group. This region has been previously shown to be specific to sensory-perceptual processing of detail that enables recognition of negative stimuli based on recollection (Mickley and Kensinger, 2008). Contrary to S2, at S3 left HC activity was more pronounced during successful in comparison with false recognition (Figure 6), which does not support previous findings where no HC activity difference was found between true and false memories (Cabeza et al., 2001). The HC has been shown to be involved in recollection rather than familiarity-based retrieval in episodic memory (Suchan et al., 2008). Moreover, the study by Suchan et al. showed consistent HC activity for recollection at immediate retrieval but also after 3- and 6-week retention intervals. In line with the hypothesis that false recognitions are more likely to be based on general features rather than on perceptual detail, these findings suggest that some brain areas such as the HC and lateral occipito-temporal areas whose activity also differed between hits and FA may reflect a dissociation between recollection and familiarity-based retrieval. Toward this end, the marked increase of FA for negative scenes in the older participants could be due to impairment in recollection processes (Comblain et al., 2004). Besides the aforementioned brain areas whose activity distinguished between successful and false recognition, the temporo-parietal junction, which is a main substrate of bottom-up attention to memory for detection of relevant information (Corbetta and Shulman, 2002; Cabeza et al., 2008) also characterized true recognition.

We did not find more activity in AMG for false recognitions in comparison with CR, and no difference was revealed in the direct comparison between hits and FA, which indicates that AMG activity is not modulated by memory accuracy. However, one study showed left AMG activity during successful recognition of negative items but not during false recognitions, supporting the hypothesis that AMG may be involved in accurate recognition of visual detail (Kensinger and Schacter, 2007). Other studies also found more AMG activity for successfully retrieved items based on recollection rather than familiarity (Sharot et al., 2004; Dolcos et al., 2005). It is possible that our analyses lacked statistical power. An alternative explanation may be that our analyses were conducted on the older group only, where gray-matter loss in left AMG was found, which may have induced dysfunction of this structure. Nonetheless, setting a more liberal threshold in our analyses (*p* < 0.05, uncorrected) indeed revealed more bilateral AMG activity for negative H than for negative FA (Table 4).

Thus, all regions highlighted in the direct comparison between successful and false recognition at S3 have been shown to have a role in retrieval based on recollection processes. A reason why these regions showed differential activity at S3 and not at S2 may be that, after long delays of retention, negative scenes are recognized based on recollection of detail, although the number of accurately recognized items has diminished. This hypothesis finds support in some previous studies showing that item recognition is based more on recollection after long than after short retention intervals, thus reflecting consolidation of these memories (Sharot et al., 2007; Sharot and Yonelinas, 2008).

## Conclusion

After delays of 1 and 3 weeks, an increased rate of false recognitions that was specific to negative scenes canceled out, in both younger and older adults, the negativity effect present for hits only. This effect was more pronounced in older adults, who nonetheless showed right AMG engagement during successful recognition of negative compared to neutral scenes, after 3 weeks delay of retention, possibly reflecting activation of relatively automatic processes during negative emotional retrieval. In the older adults, the significant higher discrimination of neutral, relative to negative scenes could be interpreted as an enhancement of the positivity effect, which overall supports Socioemotional selectivity theory. However, inclusion of positive scenes in future investigations would be more optimal to study the two sides of the effect (positive and negative, relative to neutral).

A direct comparison of activity related to successful and false recognition of negative scenes after the 3-week delay in older adults showed that regions that have been related to recollection rather than familiarity (HC and lateral occipito-temporal cortex) were more engaged for hits, indicating consolidation and true recognition of detail for negative scenes. Future studies should confirm this possibility by assessing recollection vs. familiarity in the present task situation. Another important issue raised by this study is the need to control for the semantic relatedness between targets and lures, as emotional items are in general, and particularly in the IAPS database, more likely to be grouped into categories than neutral items, which can dramatically modify memory accuracy. This issue becomes even more critical in event-related fMRI paradigms, where many items are to be used. Increased activity in VLPFC in older adults during successful recognition of negative scenes after 3 weeks retention was not related to performance or AMG activity, rejecting the hypothesis of successful compensation or emotion regulation at retrieval via VLPFC in aging. However, future studies should specifically address functional connectivity differences in aging during retrieval of emotional information between MTL and prefrontal regions. All the present findings hold for transient brain activity, and thus need to be validated by future investigations.

## Conflict of Interest Statement

The authors declare that the research was conducted in the absence of any commercial or financial relationships that could be construed as a potential conflict of interest.

## Footnotes

^1^http://www.fil.ion.ucl.ac.uk/spm

^2^http://www.mccauslandcenter.sc.edu/mricro/mricron/

## Appendix

**Table A1:** **Picture material characteristics**.

Session	Type	Emotion	*N*	Valence*	Arousal*	Living/non-living**
				Mean	Mean	Mean
S1	Target	Neutral	50	5.27	3.57	0.56
		Negative	50	2.68	5.71	0.90
	Lure	Neutral	25	5.33	3.82	0.60
		Negative	25	2.82	6.04	0.84
S2	Target	Neutral	50	5.27	3.58	0.50
		Negative	50	2.84	5.55	0.88
	Lure	Neutral	25	5.25	3.68	0.56
		Negative	25	2.67	5.76	0.84
S3	Target	Neutral	50	5.30	3.63	0.52
		Negative	50	2.64	5.64	0.86
	Lure	Neutral	25	5.34	3.73	0.56
		Negative	25	2.74	5.95	0.88

**Based on normative IAPS data. **Binary rating (0 = non-living; 1 = living)*.

*Analysis of variance on Valence with factors Session (S1, S2, S3), Type of item (target, lure), Emotion (neutral, negative): no significant main effects and interaction effects (all *F*s < 1) except main effect of Emotion (*F* = 1759.7, *p* < 0.001)*.

*Analysis of Variance on Arousal: significant main effects of Type of item (*F* = 6.8, *p* = 0.009) and Emotion (*F* = 648.3, *p* < 0.001). No significant main effect of Session (*F* = 1, *p* = 0.36) and interaction effects (all *F*s < 1)*.

*Analysis of variance on living/non-living dimension: significant main effect of Emotion (*F* = 54.4, *p* < 0.001). No other significant main or interaction effects (all *F*s < 1)*.

**Table A2:** **Neuroimaging results**.

Brain areas	BA	MNI coordinates			*t*	*k*	*p* (ROI-peak)
		*x*	*y*	*z*		
**YOUNGER GROUP**							
**Hits > misses main effect**				
HC L ROI		−16	−32	−6	3.74	33	<0.001
		−16	−4	−20	3.52	188	<0.001
HC R ROI		28	−28	−8	4.02	171	<0.001
		22	−2	−22	2.98	21	0.002
AMG L ROI		−16	−4	−18	3.33	27	0.001
AMG L ROI		−30	−2	−28	3.24	22	0.001
AMG R ROI		18	4	−16	3.49	29	<0.001
		26	−2	−28	3.09	19	0.001
Ventrolateral PFC, dorsolateral PFC L	6, 44, 45, 47	−44	40	4	6.64	1943	
Ventrolateral PFC R	45	56	30	16	3.76	73	
Paracingulate L	32	−6	34	40	4.95	170	
Cingulate posterior L	23	−2	−32	34	4.07	37	
Angular, parietal inferior L	39, 40	−42	−58	48	5.04	545	
Temporal inferior R	37	44	−56	−14	4.46	98	
Fusiform L	19	−42	−68	−12	3.92	26	
HC R		28	−28	−8	4.02	26	
HC L		−16	−32	−6	3.74	23	
Caudate L		−10	12	−2	4.33	113	
Ventral striatum R		8	2	−2	4.01	30	
Ventral striatum R		16	6	−14	4.00	20	
Cerebellum crus 1, 2 R		34	−70	−32	4.18	308	
Cerebellum lobule 6 L		−42	−52	−26	4.13	54
**Session 2 > Session 3**							
HC L ROI		−30	−14	−20	2.55	20	0.006
HC R ROI		26	−6	−22	3.66	131	<0.001
AMG R ROI		28	−4	−20	3.07	30	0.001
Occipital middle L	37, 39	−36	−68	16	4.51	302	
Calcarine R	19	28	−56	8	4.38	75	
Calcarine L	17	−8	−64	12	3.74	73	
Cuneus L	18	−8	−88	22	3.60	106	
Cingulate middle R	23	12	−28	44	4.13	54	
Temporo-parietal junction R	41, 42	52	−34	22	3.64	40	
Putamen L		−32	−8	2	4.39	119	
Putamen R		34	4	4	4.38	80	
Ventral striatum R		6	22	4	4.25	125
**Session 3 > Session 2**							
	–							
**Negative > Neutral**							
AMG L ROI		−28	2	−20	2.70	13	0.004
PFC lateral L	45, 46	−40	34	36	3.67	46	
Calcarine L	18	−8	−84	16	3.49	21
**Neutral > Negative**							
	–						
**Session × emotion interaction (neutral drop, negative rise)**							
HC L ROI		−32	−32	−12	3.65	63	<0.001
		−30	−6	−28	2.93	36	0.002
AMG L ROI		−28	−4	−24	2.80	23	0.003
Parahippocampal, fusiform L	37, 30	−32	−32	−16	4.16	220	
Parahippocampal, fusiform R	37, 30	22	−40	−12	4.10	190	
Parietal inferior L	40	−28	−46	42	4.09	82	
Parietal superior L	7	−22	−72	48	3.43	64	
Dorsomedial PFC (posterior) L	6	−22	0	52	3.67	35	
Postcentral R	3	58	−16	44	3.61	26
**Session × emotion interaction (negative drop, neutral rise)**							
	–						
**OLDER GROUP**							
**Hits > misses main effect**				
HC L ROI		−26	−26	−14	4.03	290	<0.001
HC R ROI		32	−24	−14	3.45	185	0.001
Precuneus, cingulate posterior, cuneus, retrosplenial, calcarine, lingual LR	26, 29, 30, 23, 7, 17, 18	−8	−68	22	6.56	2270	
	
Occipital mid/sup, cuneus, precuneus R	19, 7	36	−70	30	5.79	897	
Fusiform, temporal inferior/middle L	20, 37	−44	−54	−24	5.54	924	
Lingual, retrosplenial R	19, 30	22	−50	−6	3.90	80	
Temporal inferior R	20, 37	50	−46	−18	4.07	194	
Temporal superior R	38	62	2	−4	3.95	33	
Temporal superior R	22	68	−34	16	3.70	20	
Angular, parietal inferior L	39, 40	−38	−64	42	5.46	2312	
Supramarginal R	40, 2	62	−28	40	4.22	241	
Parietal inferior R	40	54	−52	48	3.91	29	
VLPFC L	45, 46	−46	44	8	4.27	149	
VLPFC L	45	−54	14	0	3.66	63	
HC L		−26	−30	−14	4.14	115	
Thalamus LR		0	−12	4	4.00	68	
Rolandic operculum R	48	48	−12	20	3.81	22	
Cerebellum crus 1 R		38	−70	−38	5.12	674	
Cerebellum lobule 6 L		−18	−74	−16	4.76	328	
Cerebellum lobule 9 L		−10	−50	−50	3.80	45
**Session 2 > Session 3**							
	–						
**Session 3 > Session 2**							
	–						
**Negative > Neutral**							
HC R ROI		26	−8	−26	2.82	43	0.003
**Neutral > Negative**							
	–
**Session × emotion**							
	–						

*ANOVAs by Age group. All results shown at *p* < 0.001 except ROI analyses at *p* < 0.0125 for HC and AMG. AMG, amygdala; BA, Brodmann Area; HC, hippocampus; L, left; MNI, Montreal Neurological Institute; PFC, prefrontal cortex; R, right; ROI, region of interest*.

**Table A3:** **Neuroimaging results**.

Brain areas	BA	MNI coordinates			*t*	*k*	*p* (ROI-peak)
		*x*	*y*	*z*		
**SESSION 2**							
**Hits > Misses main effect**							
HC L ROI		−28	−22	−18	3.85	411	<0.001
HC R ROI		32	−12	−22	4.36	514	<0.001
AMG R ROI		20	−4	−16	3.44	114	0.001
Ventrolateral PFC, dorsolateral PFC L	44, 45, 46, 47, 10, 6	−46	40	10	6.88	2400	
Angular, parietal inferior, parietal superior, temporal middle, occipital middle L	39, 40, 21, 19, 7	−36	−66	42	6.34	2330	
Cingulate middle/posterior LR	23	−2	−32	34	5.99	286	
Retrosplenial, precuneus, calcarine L	17, 18, 30, 23	−8	−56	18	5.26	460	
Temporal inferior/middle, occipital inferior, fusiform L	20, 37, 19	−42	−66	−10	5.18	1519	
Ventrolateral PFC R	45	56	30	18	4.97	158	
Temporal inferior R	20, 37	48	−56	−12	4.66	142	
Parietal superior, occipital superior R	7, 19	20	−74	42	4.44	278	
HC, parahippocampal R	28, 35, 36, 20	32	−12	−22	4.36	245	
Cingulate anterior dorsal L	32	−4	38	24	4.34	235	
Ventral striatum R	25	10	6	−8	4.26	77	
Ventral striatum L	25	−12	10	−8	4.25	91	
Thalamus LR		−2	−12	0	4.08	89	
parahippocampal, HC (posterior) R	27, 30	22	−34	−4	4.06	157	
Parietal superior R	7	24	−68	56	3.88	90	
Thalamus L		−18	−26	16	3.85	26	
Precuneus ventral R	30	12	−54	14	3.81	53	
Cerebellum crus 1, 2 R		22	−70	−32	3.62	46
**Younger > Older**							
	–
**Older > Younger**							
HC L ROI		−14	−36	8	2.87	20	0.003
Precuneus R	7	14	−70	42	4.28	62	
Thalamus L		−18	−26	16	3.99	29	
Insula L		−38	−12	18	3.89	22	
Rolandic operculum L		−36	−28	20	3.66	22	
Parietal inferior R	40	56	−52	44	3.63	28	
Temporal superior L	41	−44	−36	12	3.58	25
**Negative > Neutral**							
	–
**Neutral > Negative**							
Cerebellum lobule 8 L		−6	−64	−36	4.02	34	
Parietal inferior L	40	−30	−44	44	3.87	41	
Cerebellum lobule 6 R		24	−64	−22	3.55	40
**Age × emotion interaction**							
	–						
**SESSION 3**							
**Hits > misses main effect**							
HC L ROI		−26	−28	−12	3.19	60	0.001
HC R ROI		30	−22	−10	3.21	38	0.001
Cerebellum crus 1, 2 R		34	−72	−36	5.87	1382	
Cerebellum crus 1, 2 L		−40	−54	−28	4.78	333	
Parietal inferior, angular L	39, 40	−40	−56	50	4.67	475	
Vermis		0	−62	−34	4.09	112	
Ventrolateral PFC L	47	−48	22	−10	4.09	22	
Thalamus LR		−2	−8	6	4.01	96	
Lingual L	18	−14	−86	−14	3.97	71	
Ventrolateral PFC L	45	−48	44	−2	3.77	37
**Younger > Older**							
	–
**Older > Younger**							
HC L ROI		−34	−32	−6	3.33	77	0.001
Precuneus, cuneus, cingulate middle LR	7, 18, 23	−4	−76	24	5.00	1852	
Supramarginal, rolandic operculum, temporal superior R	48, 2, 42	52	−18	22	4.81	1112	
Temporal superior L	22	−60	−2	−2	4.70	165	
Lingual, cerebellum lobule 6 L	18	−10	−62	−16	4.59	567	
Temporal superior, supramarginal L	42, 40	−64	−32	22	4.44	159	
Occipital middle R	19	34	−68	28	4.19	60	
Supplementary motor area R	6	8	−12	66	4.15	64	
Occipital middle, angular R	39	40	−68	28	4.12	70	
Cerebellum crus 1, 2 L		−24	−82	−30	4.11	128	
Postcentral R	3	34	−22	58	3.88	66	
Cerebellum crus 1 R		20	−76	−24	3.75	111	
Postcentral R	3	34	−34	54	3.70	26	
Precentral R	6	44	−10	58	3.62	33	
Precuneus L	5	−12	−44	56	3.60	25	
Postcentral L	3	−28	−38	50	3.51	24
**Negative > Neutral**							
HC L ROI		−30	−6	−26	3.10	99	0.001
HC R ROI		34	−10	−24	3.03	53	0.002
AMG R ROI		30	−2	−26	2.93	36	0.002
Parahippocampal, fusiform R	30	20	−36	−12	4.46	32	
Dorsomedial PFC R	8	26	4	58	3.94	52	
Precuneus R	5	8	−46	68	3.89	30	
Cingulate middle L	23	−10	−22	38	3.66	27
**Neutral > Negative**							
	–
**Age × emotion interaction**							
	–						

*ANOVAs by Session. All results shown at *p* < 0.001 except ROI analyses at *p* < 0.0125 for HC and AMG. AMG, amygdala; BA, Brodmann Area; HC, hippocampus; L, left; MNI, Montreal Neurological Institute; PFC, prefrontal cortex; R, right; ROI, region of interest*.

**Table A4:** **Neuroimaging results**.

Brain areas	BA	MNI coordinates			*t*	*k*	*p* (ROI-peak)
		*x*	*y*	*z*	
**NEGATIVE**							
**Hits > misses main effect**					
HC L ROI		−30	−18	−20	4.16	309	<0.001
HC R ROI		30	−14	−22	4.38	362	<0.001
AMG L ROI		−30	−4	−22	2.89	13	0.003
AMG R ROI		32	−4	−28	3.41	73	0.001
Ventrolateral PFC L	45, 47, 44, 6	−42	42	14	6.88	3090	
Ventrolateral PFC R	45	56	30	16	5.70	275	
Dorsomedial PFC L	8	−14	20	60	4.31	55	
Cingulate anterior dorsal L	24, 32	−6	34	30	5.98	599	
Cingulate anterior dorsal R	24, 32	8	38	16	4.22	64	
Cingulate middle/posterior LR	23	−2	−32	34	5.48	373	
Cingulate middle/posterior L	23	0	−10	32	4.23	102	
Cingulate posterior, cuneus R	18, 23	16	−60	18	3.82	91	
Occipital middle, angular, parietal inferior L	19, 39, 40	−38	−72	38	5.94	1700	
Cuneus, precuneus, calcarine L	23, 7, 18	−8	−68	22	4.96	626	
Occipital superior, cuneus, precuneus R	19, 7	20	−76	42	4.48	326	
Supramarginal R	40	66	−32	32	3.65	23	
Temporal inferior L	37, 20	−44	−54	−24	4.31	668	
Temporal inferior R	37	58	−54	−10	3.58	48	
Thalamus medial LR		−4	−10	0	5.11	731	
Caudate L		−10	12	−4	5.27	264	
HC, parahippocampal L		−30	−18	−20	4.16	112	
HC, parahippocampal R		30	−14	−22	4.38	170	
Retrosplenial L	27	−14	−36	−4	3.89	68	
Cerebellum crus 1/2 R		36	−74	−36	3.81	40	
**Session 2 > Session 3**							
HC R ROI		30	−14	−22	2.95	60	0.002
**Session 3 > Session 2**							
Cerebellum lobule 6 R		−4	−64	−34	3.65	22	
**Younger > Older**							
	–						
**Older > Younger**							
HC R ROI		28	−16	−18	2.74	80	0.004
Dorsolateral PFC L	9	−28	28	34	3.93	33	
Precentral R	6	60	6	24	3.59	38	
Precuneus/cuneus R	7, 18	12	−68	36	4.50	183	
Precuneus/cuneus L	7, 18	−6	−74	26	4.00	140	
Rolandic operculum R	48	50	−12	18	4.37	196	
Rolandic operculum L	48	−60	2	6	4.23	25	
Temporal superior L	22	−60	−2	−2	4.15	24	
Temporal superior/supramarginal L	42	−64	−26	20	3.54	21	
Postcentral L	43	−52	−10	26	3.70	34	
**Session × Age interaction (younger drop, older rise)**							
Ventrolateral PFC R	45	46	46	0	3.79	35	
Ventrolateral PFC L*	45, 47	−36	36	6	3.19		
**Session × Age interaction (younger rise, older drop)**							
	–						
**NEUTRAL**							
**Hits > misses main effect**					
HC L ROI		−26	−26	−14	3.66	168	<0.001
HC R ROI		24	−28	−10	3.17	186	0.001
Ventrolateral PFC L	45, 46	−46	46	0	3.82	55	
Angular, parietal inferior L	7, 39, 40	−40	−64	44	4.71	757	
Temporal inferior R	37	46	−60	−10	4.22	117	
HC, parahippocampal L		−24	−30	−12	3.87	75	
Cerebellum crus 1 L		−16	−68	−32	3.82	36	
Cerebellum crus 1/2, lobule 6 R		14	−80	−30	5.62	966	
Cerebellum lobule 6, fusiform L	37	−40	−52	−26	5.24	952	
Vermis		−4	−60	−34	4.44	356	
Vermis		−2	−50	−12	3.74	69	
**Session 2 > Session 3**							
HC L ROI		−20	−14	−22	3.78	97	0.002
HC R ROI		22	−34	−4	3.78	97	<0.001
HC R ROI		26	−2	−26	3.27	92	0.001
AMG R ROI		30	4	−28	4.43	58	<0.001
Fusiform, parahippocampal L	30, 37	−28	−34	−18	5.05	284	
Fusiform, parahippocampal R	30, 37	22	−36	−12	4.21	89	
Fusiform, parahippocampal R	20	36	−32	−20	4.01	32	
Temporal inferior L	20	−46	−46	−14	3.62	26	
AMG R		30	4	−28	4.43	28	
Parietal superior L	7	−20	−72	54	3.99	43	
Occipital superior L	18	−24	−92	26	3.85	33	
Putamen L		−30	−12	2	4.02	35	
Postcentral R	3	40	−18	34	3.66	35	
Postcentral L	4	−54	−18	50	3.43	22	
**Session 3 > Session 2**							
	–						
**Younger > Older**							
	–						
**Older > Younger**							
HC L ROI		−36	−34	−6	3.16	59	0.001
Precuneus R	5	10	−46	56	4.52	109	
Parietal inferior, supramarginal, angular R	40, 39	60	−44	38	4.23	284	
Supramarginal R	48	52	−18	26	4.21	135	
Calcarine, lingual L	17	−8	−66	2	4.15	105	
Rolandic operculum L	48	−30	−30	14	3.81	21	
Postcentral R	3	30	−40	54	4.30	88	
Postcentral R	3	48	−18	52	3.81	21	
**Session × Age interaction**							
	–						

*ANOVAs by Emotion. All results shown at *p* < 0.001 except ROI analyses at *p* < 0.0125 for HC and AMG. *Left ventrolateral PFC significant at *p* < 0.005. AMG, amygdala; BA, Brodmann Area; HC, hippocampus; L, left; MNI, Montreal Neurological Institute; PFC, prefrontal cortex; R, right; ROI, region of interest*.

## References

[B1] Anderson M. C. Weaver C. (2009). “Inhibitory control over action and memory,” in Encyclopedia of Neuroscience, ed. Squire L. R. (Oxford: Academic Press), 153–163

[B2] Andreasen N. C. O’Leary D. S. Arndt S. Cizadlo T. Hurtig R. Rezai K. Watkins G. L. Ponto L. L. Hichwa R. D. (1995). Short-term and long-term verbal memory: a positron emission tomography study. Proc. Natl. Acad. Sci. U.S.A. 92, 5111–5115 10.1073/pnas.92.11.5111 7761457 PMC41858

[B3] Aron A. R. Fletcher P. C. Bullmore E. T. Sahakian B. J. Robbins T. W. (2003). Stop-signal inhibition disrupted by damage to right inferior frontal gyrus in humans. Nat. Neurosci. 6, 115–116 10.1038/nn1003 12536210

[B4] Aron A. R. Robbins T. W. Poldrack R. A. (2004). Inhibition and the right inferior frontal cortex. Trends Cogn. Sci. 8, 170–177 10.1016/j.tics.2004.02.010 15050513

[B5] Ashburner J. (2007). A fast diffeomorphic image registration algorithm. Neuroimage 38, 95–113 10.1016/j.neuroimage.2007.07.007 17761438

[B6] Bastin C. van der Linden M. (2003). The contribution of recollection and familiarity to recognition memory: a study of the effects of test format and aging. Neuropsychology 17, 14–24 10.1037/0894-4105.17.1.14 12597069

[B7] Bosshardt S. Degonda N. Schmidt C. F. Boesiger P. Nitsch R. M. Hock C. Henke K. (2005a). One month of human memory consolidation enhances retrieval-related hippocampal activity. Hippocampus 15, 1026–1040 10.1002/hipo.20105 16015623

[B8] Bosshardt S. Schmidt C. F. Jaermann T. Degonda N. Boesiger P. Nitsch R. M. Hock C. Henke K. (2005b). Effects of memory consolidation on human hippocampal activity during retrieval. Cortex 41, 486–498 10.1016/S0010-9452(08)70189-8 16042025

[B9] Cabeza R. Ciaramelli E. Olson I. R. Moscovitch M. (2008). The parietal cortex and episodic memory: an attentional account. Nat. Rev. Neurosci. 9, 613–625 10.1038/nrn2459 18641668 PMC2692883

[B10] Cabeza R. Locantore J. K. Anderson N. D. (2003). Lateralization of prefrontal activity during episodic memory retrieval: evidence for the production-monitoring hypothesis. J. Cogn. Neurosci. 15, 249–259 10.1162/089892903321208187 12676062

[B11] Cabeza R. Rao S. M. Wagner A. D. Mayer A. R. Schacter D. L. (2001). Can medial temporal lobe regions distinguish true from false? An event-related functional MRI study of veridical and illusory recognition memory. Proc. Natl. Acad. Sci. U.S.A. 98, 4805–4810 10.1073/pnas.081082698 11287664 PMC31915

[B12] Carstensen L. L. Isaacowitz D. M. Charles S. T. (1999). Taking time seriously. A theory of socioemotional selectivity. Am. Psychol. 54, 165–181 10.1037/0003-066X.54.3.165 10199217

[B13] Charles S. T. Mather M. Carstensen L. L. (2003). Aging and emotional memory: the forgettable nature of negative images for older adults. J. Exp. Psychol. Gen. 132, 310–324 10.1037/0096-3445.132.2.310 12825643

[B14] Comblain C. D’Argembeau A. van der Linden M. Aldenhoff L. (2004). The effect of ageing on the recollection of emotional and neutral pictures. Memory 12, 673–684 10.1080/09658210344000477 15724356

[B15] Corbetta M. Shulman G. L. (2002). Control of goal-directed and stimulus-driven attention in the brain. Nat. Rev. Neurosci. 3, 201–215 10.1038/nrn755 11994752

[B16] Davis S. W. Kragel J. E. Madden D. J. Cabeza R. (2012). The architecture of cross-hemispheric communication in the aging brain: linking behavior to functional and structural connectivity. Cereb. Cortex 22, 232–242 10.1093/cercor/bhr123 21653286 PMC3236798

[B17] Denburg N. L. Buchanan T. W. Tranel D. Adolphs R. (2003). Evidence for preserved emotional memory in normal older persons. Emotion 3, 239–253 10.1037/1528-3542.3.3.239 14498794

[B18] Dolan R. J. Lane R. Chua P. Fletcher P. (2000). Dissociable temporal lobe activations during emotional episodic memory retrieval. Neuroimage 11, 203–209 10.1016/S1053-8119(00)91136-4 10694462

[B19] Dolcos F. Denkova E. Dolcos S. (2012). Neural correlates of emotional memories: a review of evidence from brain imaging studies. Accepted for publication in the special issue on “Recent advances of functional neuroimaging studies on episodic memories.” Psychologia (in press).

[B20] Dolcos F. LaBar K. S. Cabeza R. (2005). Remembering one year later: role of the amygdala and the medial temporal lobe memory system in retrieving emotional memories. Proc. Natl. Acad. Sci. U.S.A. 102, 2626–2631 10.1073/pnas.0409848102 15703295 PMC548968

[B21] Dupont S. Samson Y. van de Moortele P. F. Samson S. Poline J. B. Adam C. Lehéricy S. Le Bihan D. Baulac M. (2001). Delayed verbal memory retrieval: a functional MRI study in epileptic patients with structural lesions of the left medial temporal lobe. Neuroimage 14, 995–1003 10.1006/nimg.2001.0908 11697931

[B22] Ebner N. C. Johnson M. K. Fischer H. (2012). Neural mechanisms of reading facial emotions in young and older adults. Front. Psychol. 3:223 10.3389/fpsyg.2012.00223 22798953 PMC3394436

[B23] Fischer H. Nyberg L. Bäckman L. (2010). Age-related differences in brain regions supporting successful encoding of emotional faces. Cortex 46, 490–497 10.1016/j.cortex.2009.05.011 19560133

[B24] Gavazzeni J. Andersson T. Bäckman L. Wiens S. Fischer H. (2012). Age, gender, and arousal in recognition of negative and neutral pictures 1 year later. Psychol. Aging. 10.1037/a0027946 [Epub ahead of print]. 22506603

[B25] Howard M. W. Bessette-Symons B. Zhang Y. Hoyer W. J. (2006). Aging selectivity impairs recollection in recognition memory for pictures: evidence from modeling and receiver operating characteristic curves. Psychol. Aging 21, 96–106 10.1037/0882-7974.21.1.96 16594795 PMC1749613

[B26] Howe M. L. Candel I. Otgaar H. Malone C. Wimmer M. C. (2010). Valence and the development of immediate and long-term false memory illusions. Memory 18, 58–75 10.1080/09658210903476514 20391177

[B27] Huijbers W. Pennartz C. M. A. Rubin D. C. Daselaar S. M. (2011). Imagery and retrieval of auditory and visual information: neural correlates of successful and unsuccessful performance. Neuropsychologia 49, 1730–1740 10.1016/j.neuropsychologia.2011.02.051 21396384

[B28] Janzen G. Jansen C. van Turennout M. (2008). Memory consolidation of landmarks in good navigators. Hippocampus 18, 40–47 10.1002/hipo.20364 17924521

[B29] Kalpouzos G. Nyberg L. (2010). “Asymmetry of memory in the brain,” in The Two Halves of the Brain: Information Processing in the Cerebral Hemispheres, eds Hugdahl K. Westerhausen R. (Cambridge, MA: MIT Press), 499–530

[B30] Kalpouzos G. Nyberg L. (2012). “Multimodal neuroimaging in normal aging: structure-function interactions,” in Memory and Aging: Current Issues and Future Directions, eds Naveh-Benjamin M. Ohta N. (Hove: Psychology Press), 273–304

[B31] Kalpouzos G. Persson J. Nyberg L. (2012). Local brain atrophy accounts for functional activity differences in normal aging. Neurobiol. Aging 33, 623.e1–623.e13 10.1016/j.neurobiolaging.2011.02.021 21524432

[B32] Keightley M. L. Chiew K. S. Anderson J. A. E. Grady C. L. (2011). Neural correlates of recognition memory for emotional faces and scenes. Soc. Cogn. Affect. Neurosci. 6, 24–37 10.1093/scan/nsq003 20194514 PMC3023078

[B33] Kensinger E. Corkin S. (2004). The effects of emotional content and aging on false memories. Cogn. Affect. Behav. Neurosci. 4, 1–9 10.3758/CABN.4.1.1 15259885

[B34] Kensinger E. A. (2007). Negative emotion enhances memory accuracy. Curr. Dir. Psychol. Sci. 16, 213–218 10.1111/j.1467-8721.2007.00506.x

[B35] Kensinger E. A. (2012). “Emotion-memory interactions in older adulthood,” in Memory and Aging: Current Issues and Future Directions, eds Naveh-Benjamin M. Ohta N. (Hove: Psychology Press), 215–244

[B36] Kensinger E. A. Brierley B. Medford N. Growdon J. H. Corkin S. (2002). Effects of normal aging and Alzheimer’s disease on emotional memory. Emotion 2, 118–134 10.1037/1528-3542.2.2.118 12899186

[B37] Kensinger E. A. Schacter D. L. (2005). Retrieving accurate and distorted memories: neuroimaging evidence for effects of emotion. Neuroimage 27, 167–177 10.1016/j.neuroimage.2005.03.038 15919215

[B38] Kensinger E. A. Schacter D. L. (2007). Remembering the specific visual details of presented objects: neuroimaging evidence for effects of emotion. Neuropsychologia 45, 2951–2962 10.1016/j.neuropsychologia.2007.05.024 17631361

[B39] Kensinger E. A. Schacter D. L. (2008). Neural processes supporting young and older adults’ emotional memories. J. Cogn. Neurosci. 20, 1161–1173 10.1162/jocn.2008.20080 18284340

[B40] Kuhl B. A. Dudukovic N. M. Kahn I. Wagner A. D. (2007). Decreased demands on cognitive control reveal the neural processing benefits of forgetting. Nat. Neurosci. 10, 908–914 10.1038/nn1918 17558403

[B41] Kuhl B. A. Kahn I. Dudukovic N. M. Wagner A. D. (2008). Overcoming suppression in order to remember: contributions from anterior cingulate and ventrolateral prefrontal cortex. Cogn. Affect. Behav. Neurosci. 8, 211–221 10.3758/CABN.8.2.211 18589510 PMC2490713

[B42] LaBar K. S. Phelps E. A. (1998). Arousal-mediated memory consolidation: role of the medial temporal lobe in humans. Psychol. Sci. 9, 490–493 10.1111/1467-9280.00090

[B43] Lang P. J. Bradley M. M. Cuthbert B. N. (2008). International Affective Picture System (IAPS): Affective Ratings of Pictures and Instruction Manual. Technical Report A-8. Gainesville, FL: University of Florida

[B44] Leshikar E. D. Gutchess A. H. Hebrank A. C. Sutton B. P. Park D. C. (2010). The impact of increased relational encoding demands on frontal and hippocampal function in older adults. Cortex 46, 507–521 10.1016/j.cortex.2009.07.011 19709652 PMC2826535

[B45] Maldjian J. A. Laurienti P. J. Burdette J. B. Kraft R. A. (2003). An automated method for neuroanatomic and cytoarchitectonic atlas-based interrogation of fMRI data sets. Neuroimage 19, 1233–1239 10.1016/S1053-8119(03)00169-1 12880848

[B46] Maratos E. J. Allan K. Rugg M. D. (2000). Recognition memory for emotionally negative and neutral words: an ERP study. Neuropsychologia 38, 1452–1465 10.1016/S0028-3932(00)00061-0 10906371

[B47] Marchewka A. Brechmann A. Nowicka A. Jednoróg K. Scheich H. Grabowska A. (2008). False recognition of emotional stimuli is lateralised in the brain: an fMRI study. Neurobiol. Learn. Mem. 90, 280–284 10.1016/j.nlm.2008.01.012 18329298

[B48] Mather M. (2008). “Why memories may become more positive as people age,” in Memory and Emotion: Interdisciplinary Perspectives, eds Uttl B. Ohta N. Siegenthaler A. L. (Oxford: Blackwell Publishing), 135–158

[B49] Mather M. (2012). The emotion paradox in the aging brain. Ann. N. Y. Acad. Sci. 1251, 33–49 10.1111/j.1749-6632.2012.06471.x 22409159 PMC3395773

[B50] Mather M. Carstensen L. L. (2005). Aging and motivated cognition: the positivity effect in attention and memory. Trends Cogn. Sci. (Regul. Ed.) 9, 496–502 10.1016/j.tics.2005.08.005 16154382

[B51] Mickley K. Kensinger E. (2008). Emotional valence influences the neural correlates associated with remembering and knowing. Cogn. Affect. Behav. Neurosci. 8, 143–152 10.3758/CABN.8.2.143 18589505

[B52] Murphy N. A. Isaacowitz D. M. (2008). Preferences for emotional information in older and younger adults: a meta-analysis of memory and attention tasks. Psychol. Aging 23, 263–286 10.1037/0882-7974.23.2.263 18573002

[B53] Murty V. P. Ritchey M. Adcock R. A. LaBar K. S. (2010). fMRI studies of successful emotional memory encoding: a quantitative meta-analysis. Neuropsychologia 48, 3459–3469 10.1016/j.neuropsychologia.2010.07.030 20688087 PMC2949536

[B54] Murty V. P. Sambataro F. Das S. Tan H. Y. Callicott J. H. Goldberg T. E. Meyer-Lindenberg A. Weinberger D. R. Mattay V. S. (2009). Age-related alterations in simple declarative memory and the effect of negative stimulus valence. J. Cogn. Neurosci. 21, 1920–1933 10.1162/jocn.2009.21130 18823239 PMC2757312

[B55] Nolde S. F. Johnson M. K. Raye C. L. (1998). The role of prefrontal cortex during tests of episodic memory. Trends Cogn. Sci. (Regul. Ed.) 2, 399–406 10.1016/S1364-6613(98)01233-9 21227255

[B56] Otani H. Libkuman T. M. Widner R. L. Graves E. I. (2007). Memory for emotionally arousing stimuli: a comparison of younger and older adults. J. Gen. Psychol. 134, 23–42 10.3200/GENP.134.1.23-42 17283853

[B57] Pierce B. H. Kensinger E. A. (2011). Effects of emotion on associative recognition: valence and retention interval matter. Emotion 11, 139–144 10.1037/a0021287 21401233 PMC3106271

[B58] Pollock J. W. Khoja N. Kaut K. P. Lien M. Allen P. (2012). Electrophysiological evidence for adult age-related sparing and decrements in emotion perception and attention. Front. Integr. Neurosci. 6:60 10.3389/fnint.2012.00060 22936901 PMC3426158

[B59] Prull M. W. Dawes L. L. Martin A. M. III Rosenberg H. F. Light L. L. (2006). Recollection and familiarity in recognition memory: adult age differences and neuropsychological test correlates. Psychol. Aging 21, 107–118 10.1037/0882-7974.21.1.107 16594796

[B60] Putcha D. Brickhouse M. O’Keefe K. Sullivan C. Rentz D. Marshall G. Dickerson B. Sperling R. (2011). Hippocampal hyperactivation associated with cortical thinning in Alzheimer’s disease signature regions in non-demented elderly adults. J. Neurosci. 31, 17680–17688 10.1523/JNEUROSCI.4740-11.2011 22131428 PMC3289551

[B61] Rajah M. N. D’Esposito M. (2005). Region-specific changes in prefrontal function with age: a review of PET and fMRI studies on working and episodic memory. Brain 128, 1964–1983 10.1093/brain/awh608 16049041

[B62] Reed A. E. Carstensen L. L. (2012). The theory behind the age-related positivity effect. Front. Psychol. 3:339 10.3389/fpsyg.2012.00339 PMC3459016 23060825

[B63] Sabatinelli D. Fortune E. E. Li Q. Siddiqui A. Krafft C. Oliver W. T. Beck S. Jeffries J. (2011). Emotional perception: meta-analyses of face and natural scene processing. Neuroimage 54, 2524–2533 10.1016/j.neuroimage.2010.10.011 20951215

[B64] Sharot T. Delgado M. R. Phelps E. A. (2004). How emotion enhances the feeling of remembering. Nat. Neurosci. 7, 1376–1380 10.1038/nn1353 15558065

[B65] Sharot T. Phelps E. (2004). How arousal modulates memory: disentangling the effects of attention and retention. Cogn. Affect. Behav. Neurosci. 4, 294–306 10.3758/CABN.4.3.294 15535165

[B66] Sharot T. Verfaellie M. Yonelinas A. P. (2007). How emotion strengthens the recollective experience: a time-dependent hippocampal process. PLoS ONE 2, e1068 10.1371/journal.pone.0001068 17971848 PMC2031918

[B67] Sharot T. Yonelinas A. P. (2008). Differential time-dependent effects of emotion on recollective experience and memory for contextual information. Cognition 106, 538–547 10.1016/j.cognition.2007.03.002 17451666

[B68] Snodgrass J. G. Corwin J. (1988). Pragmatics of measuring recognition memory: applications to dementia and amnesia. J. Exp. Psychol. Gen. 117, 34–50 10.1037/0096-3445.117.1.34 2966230

[B69] Spaniol J. Davidson P. S. R. Kim A. S. N. Han H. Moscovitch M. Grady C. L. (2009). Event-related fMRI studies of episodic encoding and retrieval: meta-analyses using activation likelihood estimation. Neuropsychologia 47, 1765–1779 10.1016/j.neuropsychologia.2009.02.028 19428409

[B70] Spaniol J. Voss A. Grady C. L. (2008). Aging and emotional memory: cognitive mechanisms underlying the positivity effect. Psychol. Aging 23, 859–872 10.1037/a0014218 19140656

[B71] St Jacques P. L. Bessette-Symons B. Cabeza R. (2009a). Functional neuroimaging studies of aging and emotion: fronto-amygdalar differences during emotional perception and episodic memory. J. Int. Neuropsychol. Soc. 15, 819–825 10.1017/S1355617709990439 19703320 PMC3633489

[B72] St Jacques P. L. Dolcos F. Cabeza R. (2009b). Effects of aging on functional connectivity of the amygdala for subsequent memory of negative pictures. Psychol. Sci. 20, 74–84 10.1111/j.1467-9280.2008.02258.x 19152542 PMC3633516

[B73] Stark C. E. L. Squire L. R. (2000). fMRI activity in the medial temporal lobe during recognition memory as a function of study-test interval. Hippocampus 10, 329–337 10.1002/1098-1063(2000)10:3<329::AID-HIPO13>3.0.CO;2-Z 10902902

[B74] Suchan B. Gayk A. E. Schmid G. Köster O. Daum I. (2008). Hippocampal involvement in recollection but not familiarity across time: a prospective study. Hippocampus 18, 92–98 10.1002/hipo.20371 17932973

[B75] Takashima A. Nieuwenhuis I. L. C. Jensen O. Talamini L. M. Rijpkema M. Fernández G. (2009). Shift from hippocampal to neocortical centered retrieval network with consolidation. J. Neurosci. 29, 10087–10093 10.1523/JNEUROSCI.0799-09.2009 19675242 PMC6664975

[B76] Takashima A. Petersson K. M. Rutters F. Tendolkar I. Jensen O. Zwarts M. J. McNaughton B. L. Fernández G. (2006). Declarative memory consolidation in humans: a prospective functional magnetic resonance imaging study. Proc. Natl. Acad. Sci. U.S.A. 103, 756–761 10.1073/pnas.0507774103 16407110 PMC1334654

[B77] Tulving E. Kapur S. Craik F. I. Moscovitch M. Houle S. (1994). Hemispheric encoding/retrieval asymmetry in episodic memory: positron emission tomography findings. Proc. Natl. Acad. Sci. U.S.A. 91, 2016–2020 10.1073/pnas.91.6.2012 8134342 PMC43300

[B78] Tzourio-Mazoyer N. Landeau B. Papathanassiou D. Crivello F. Etard O. Delcroix N. Mazoyer B. Joliot M. (2002). Automated anatomical labeling of activations in SPM using a macroscopic anatomical parcellation of the MNI MRI single-subject brain. Neuroimage 15, 273–289 10.1006/nimg.2001.0978 11771995

[B79] Viskontas I. V. Carr V. A. Engel S. A. Knowlton B. J. (2009). The neural correlates of recollection: hippocampal activation declines as episodic memory fades. Hippocampus 19, 265–272 10.1002/hipo.20503 18830998

[B80] Waring J. D. Kensinger E. A. (2009). Effects of emotional valence and arousal upon memory trade-offs with aging. Psychol. Aging 24, 412–422 10.1037/a0015526 19485658

[B81] Weymar M. Löw A. Hamm A. O. (2011). Emotional memories are resilient to time: evidence from the parietal ERP old/new effect. Hum. Brain Mapp. 32, 632–640 10.1002/hbm.21051 21391253 PMC6870483

[B82] Winocur G. Moscovitch M. (2011). Memory transformation and systems consolidation. J. Int. Neuropsychol. Soc. 17, 1–15 10.1017/S1355617711000804 21729403

